# Secondary damage and neuroinflammation in the spinal dorsal horn mediate post-thalamic hemorrhagic stroke pain hypersensitivity: SDF1-CXCR4 signaling mediation

**DOI:** 10.3389/fnmol.2022.911476

**Published:** 2022-08-12

**Authors:** Ting Liang, Xue-Feng Chen, Yan Yang, Fei Yang, Yang Yu, Fan Yang, Xiao-Liang Wang, Jiang-Lin Wang, Wei Sun, Jun Chen

**Affiliations:** ^1^Institute for Biomedical Sciences of Pain, Tangdu Hospital, The Fourth Military Medical University, Xi’an, China; ^2^Key Laboratory of Brain Stress and Behavior, People’s Liberation Army, Xi’an, China; ^3^Department of Anesthesiology and Perioperative Medicine, Clinical Medical College (900 Hospital of the Joint Logistic Support Force), Fujian Medical University, Fuzhou, China; ^4^Department of Pain Medicine, The Affiliated Hospital, Southwest Medical University, Luzhou, China

**Keywords:** central post-stroke pain, thalamic hemorrhagic stroke, hyperalgesia, retrograde degeneration, neuroinflammation, glial cells, SDF1-CXCR4, spinal dorsal horn

## Abstract

Central post-stroke pain (CPSP) is an intractable neuropathic pain, which can be caused by primary lesion of central somatosensory system. It is also a common sequelae of the thalamic hemorrhagic stroke (THS). So far, the underlying mechanisms of CPSP remain largely unknown. Our previous studies have demonstrated that SDF1-CXCR4 signaling in the hemorrhagic region contributes to the maintenance of the THS pain hypersensitivity *via* mediation of the thalamic neuroinflammation. But whether the spinal dorsal horn, an initial point of spinothalamic tract (STT), suffers from retrograde axonal degeneration from the THS region is still unknown. In this study, neuronal degeneration and loss in the spinal dorsal horn were detected 7 days after the THS caused by intra-thalamic collagenase (ITC) injection by immunohistochemistry, TUNEL staining, electron microscopy, and extracellular multi-electrode array (MEA) recordings, suggesting the occurrence of secondary apoptosis and death of the STT projecting neuronal cell bodies following primary THS *via* retrograde axonal degeneration. This retrograde degeneration was accompanied by secondary neuroinflammation characterized by an activation of microglial and astrocytic cells and upregulation of SDF1-CXCR4 signaling in the spinal dorsal horn. As a consequence, central sensitization was detected by extracellular MEA recordings of the spinal dorsal horn neurons, characterized by hyperexcitability of both wide dynamic range and nociceptive specific neurons to suprathreshold mechanical stimuli. Finally, it was shown that suppression of spinal neuroinflammation by intrathecal administration of inhibitors of microglia (minocycline) and astrocytes (fluorocitrate) and antagonist of CXCR4 (AMD3100) could block the increase in expression levels of Iba-1, GFAP, SDF1, and CXCR4 proteins in the dorsal spinal cord and ameliorate the THS-induced bilateral mechanical pain hypersensitivity, implicating that, besides the primary damage at the thalamus, spinal secondary damage and neuroinflammation also play the important roles in maintaining the central post-THS pain hypersensitivity. In conclusion, secondary neuronal death and neuroinflammation in the spinal dorsal horn can be induced by primary thalamic neural damage *via* retrograde axonal degeneration process. SDF1-CXCR4 signaling is involved in the mediation of secondary spinal neuroinflammation and THS pain hypersensitivity. This finding would provide a new therapeutic target for treatment of CPSP at the spinal level.

## Introduction

Stroke is the second major cause of death and the third-leading cause of adult disability worldwide (GBD 2019 Stroke Collaborators, 2021; Owolabi et al., 2022). Although the mortality rate of stroke has been declined over the past two decades, years with disability due to stroke is increasing (Kimura, 2020). One of the most troublesome sequelae of stroke is CPSP, which can be caused by both ischemic and hemorrhagic lesions of the somatosensory system (Klit et al., 2009; Kumar et al., 2009a; Kumar and Soni, 2009b). CPSP is a typical chronic central neuropathic pain, which has been listed in the pain categories of 11th revision of the International Classification of Diseases (ICD-11) (Scholz et al., 2019). Generally, it is believed that the anatomical, neurochemical, and neuroinflammatory changes caused by the central somatosensory system lesion or diseases can trigger neuronal hyperexcitability or central sensitization, resulting in chronic CPSP (Kuan et al., 2015; Yang et al., 2017a; Li et al., 2018; Wan et al., 2020). The symptoms of patients with CPSP can be continuous or intermittent unpleasant experiences described as burning, cold, lancinating, throbbing, pressing, or stinging-like pain (Ofek and Defrin, 2007; Klit et al., 2009; Park et al., 2021). CPSP can be partially relieved by the first-line pharmacological therapies using tricyclic antidepressants (amitriptyline and imipramine), the SSRI (fluoxetine), and the anticonvulsants (gabapentin and pregabalin) (Choi et al., 2021a). Moreover, non-pharmacological therapies such as transcranial direct current stimulation and deep brain stimulation have also been used (Xu et al., 2020). However, 50–60% patients with CPSP remain intractable to any of the above approaches due to poor knowledge of the underlying mechanisms of CPSP.

To get an understanding of the underlying mechanisms of CPSP, animal models with high reproducibility and clinical relevance are critical (De Vloo et al., 2017). In the existing animal models for studying CPSP, the experimental thalamic hemorrhagic stroke (THS) model induced by intra-thalamic collagenase IV (ITC) injection in both rats and mice has been demonstrated to be one with relatively high reproducibility, injury region specificity, and pharmacologically anticonvulsant (gabapentin) and antineuroinflammation reversibility (Wasserman and Koeberle, 2009; Yang et al., 2014, 2016, 2017a; Cai et al., 2018; Huang et al., 2020; Fu et al., 2021). Besides the changes surrounding the thalamic hemorrhagic region (Yang et al., 2017a; Cai et al., 2018; Huang et al., 2020; Fu et al., 2021), changes in the ACC, secondary somatosensory cortex, posterior insular cortex, hippocampus, and amygdala have also been demonstrated to be involved in the processing of CPSP (Kuan et al., 2015; Nagasaka et al., 2019; Infantino et al., 2022). Although the THS-induced pain hypersensitivity has been shown to be ameliorated by some antidepressants (amitriptyline, imipramine, fluoxetine) and anticonvulsant (gabapentin but not carbamazepine) in rodents (Yang et al., 2014; Shyu et al., 2021), drug tolerance may occur after repeated administration of gabapentin, for example, due to off-target of α2δ1 subunits of voltage-gated calcium channels (Yang et al., 2014, 2016). Actually, the THS-induced CPSP has been known to be initiated by HIF-1α but maintained by local neuroinflammation mediated by glial–glial (microglia and astrocytes) and glial–neuronal interactions *via* SDF1-CXCR4 signaling in rats (Yang et al., 2017a). SDF1-CXCR4 signaling-mediated neuroinflammation (Timotijević et al., 2012) is critical to both inflammatory and neuropathic pain across the whole somatosensory system from the dorsal root ganglion (DRG) (Yang et al., 2015, 2017b; Bai et al., 2016; Qiu et al., 2016), to the spinal dorsal horn (Shen et al., 2014; Bai et al., 2016; Li et al., 2016, 2019; Luo et al., 2016a,b; Hu et al., 2017, 2020; Xing et al., 2017; Xu et al., 2017; Liu et al., 2019), and to the thalamus (Yang et al., 2017a) after tissue and nerve injury.

It is known that paw withdrawal reflex to both heat and mechanical noxious stimuli is mainly mediated by the spinally organized flexion reflex circuitry (You et al., 2003; Li and Chen, 2004; Li et al., 2008). However, how the THS-induced neural damage facilitates the spinally organized flexion reflex remains unknown. One possibility is that primary thalamic damage can cause retrograde axonal degeneration of the STT projecting fiber terminals, resulting in secondary spinal STT neuronal cell body death and neuroinflammation. Some previous reports support our presumption: (1) diffuse tensor tractography (DTT) showed likely damage of STT fibers after CPSP (Jang et al., 2019; Park et al., 2021); (2) involvement of spinal NOS signal in the development of bilateral carotid artery occlusion induced CPSP (Matsuura et al., 2019); (3) blockade of peripheral sensory input resulted in amelioration of CPSP (Haroutounian et al., 2018; Choi et al., 2021b). To address the above question, this study was designed to examine whether secondary neuronal death and neuroinflammation occur in the spinal dorsal horn after an establishment of the THS-induced pain hypersensitivity in rats, and if any, whether i.t. antineuroinflammation treatment can relieve the ITC-induced THS pain hypersensitivity?

## Materials and methods

### Animals

Male Sprague–Dawley rats weighing 180–230 g were purchased from the Laboratory Animal Center of Fourth Military Medical University (FMMU). All animals were housed in groups of 4–6 rats per cage with food and water *ad libitum* and maintained under standard laboratory condition (12-h light/dark cycle, lights on 08:00–20:00, temperature 22–26°C, air humidity 40–60%). This study was approved by the Institutional Animal Care and Use Committee of the FMMU (#20190150) and fully in accordance with the recommendations of the ARRIVE guidelines (Kilkenny et al., 2010), the United Kingdom Animals (Scientific Procedures) Act 1986 and associated guidelines, the EU Directive 2010/63/EU for animal experiments, the National Institutes of Health guide for the care and use of laboratory animals (NIH Publications no. 8023, revised 1978), and the ethical guidelines for the investigations of experimental pain in conscious animals of the International Association for the Study of Pain were also critically followed (Zimmermann, 1983). The number and suffering of animals were greatly minimized as required.

### The central post-stroke pain model

**FIGURE 1:**
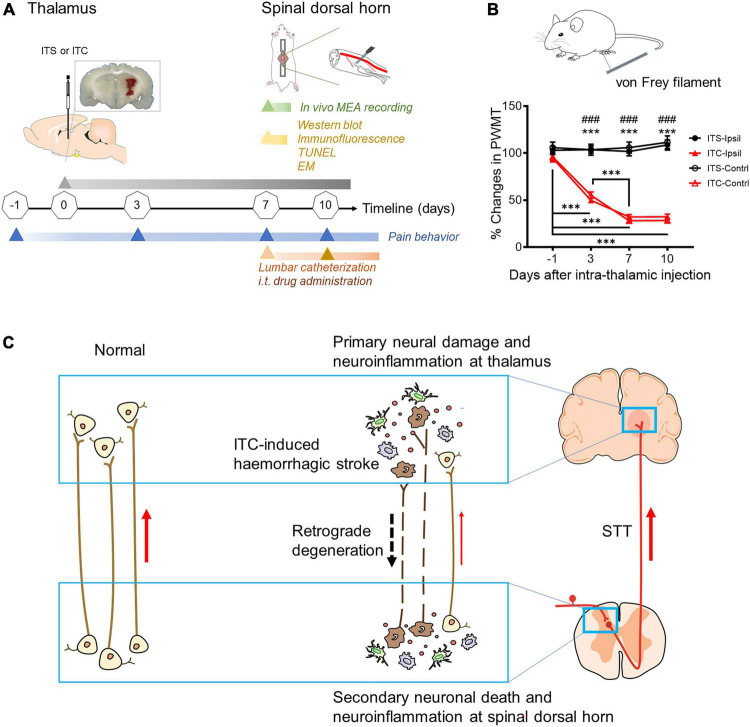
Diagram of experimental timeline and hypothesis of this study. **(A)** Experimental timelines. EM, electron microscopy; i.t, intrathecal; ITC, intra-thalamic collagenase IV injection; ITS, intra-thalamic saline injection; MEA, multi-electrode array; TUNEL, terminal deoxynucleotidyl transferase-mediated nick end labeling; **(B)** time courses of bilateral mechanical pain hypersensitivity induced by left ITC with ITS as control. Contrl, contralateral; Ipsil, ipsilateral; PWMT, paw withdrawal mechanical threshold; ***p < 0.001 Day 3, Day 7, Day 10 vs. one day prior to surgery; ###*p* < 0.001 ITC-Ipsil vs. ITS-Ipsil; ***p < 0.001 ITC-Contrl vs. ITS-Contrl; *n* = 44 rats for ITS, *n* = 59 rats for ITC. **(C)** Hypothetic scheme proposes that post-thalamic hemorrhagic stroke (THS) pain hypersensitivity should be mediated by the THS-induced secondary neural damage and neuroinflammation in the spinal dorsal horn through retrograde axonal degeneration.

Rats were randomly divided into two groups for establishment of the CPSP model with ITC-induced thalamic hemorrhage: (1) rats receiving ITS; (2) rats receiving ITC. Surgery was operated on the rats as described previously (Yang et al., 2014, 2016, 2017a). Rats were securely fixed in a stereotaxic instrument (RWD, China) after being anesthetized with sodium pentobarbital (50 mg/kg, i.p.). After a midline incision, an opening was made in the right or left skull with a dental drill. An 0.5-μl microsyringe filled with collagenase type IV (Sigma–Aldrich China, Shanghai) or saline was made into the region including VBC and posterior thalamic nucleus (Po) of the right or left thalamus (bregma –2.28 to –3.48 mm anteroposterior; 3.0–3.6 mm lateral to the midline, and 6.2 mm ventral from the brain surface) according to the stereotaxic coordinates (Paxinos and Watson, 2005). The microsyringe was lowered into the target region and stayed for 5 min before slow administration of ITC (0.025 IU collagenase dissolved in 0.25 μl of saline) or ITS (0.25 μl of saline) over a period of 10 min (Figure 1A). The syringe remained for 5 min after each injection before the needle was slowly withdrawn. The skin was disinfected after being sutured. All rats were allowed to recover in individual cages for at least 7 days (Figure 1A).

### Measurement of mechanical pain sensitivity

The rats were acclimated 30 min before the testing in an opaque plexiglass box (20 cm × 20 cm × 25 cm) placed on a metal mesh (1 cm × 1 cm) that fully exposed to the rats’ feet. Ascending graded individual von Frey monofilaments with 15 bending forces ranging from 1.0 to 60 g were used for the measurement of mechanical pain sensitivity. Each von Frey filament was applied to the bilateral plantar surface of hind paws ten times (once every several seconds) before and 3, 7, and 10 days after ITC or ITS. For pharmacological evaluations, von Frey tests were conducted 3 days after i.t. catheterization for 2, 4, 6, 8, 24, and 48 h (10 days after ITC). The minimum bending force value of the von Frey filament that enabled 50% occurrence of withdrawal reflex was marked as the PWMT (g). For details, refer to Yu et al. (2019).

### Implantation of intrathecal catheters and administration of drug

The surgery was performed according to the method described previously (Yang et al., 2015). Rats with ITS or ITC were anesthetized with sodium pentobarbital and then placed in prone position to fully expose the lumbar intervertebral space 7 days after ITS or ITC (Figure 1A). The operative area was disinfected and covered with sterile drapes. The L5-6 vertebrae was fully exposed by a 2- to 3-cm longitudinal incision parallel to the spine and blunt dissection of paraspinal muscles. A flattened syringe needle was used to drill into the punctured intervertebral space slowly. The proper placement of i.t. injection into L5-6 intervertebral space was confirmed by tail flicking or hind paw retraction. Thereafter, the PE-10 catheter filled with sterile saline was inserted through the slit to reach the lumbar enlargement. The catheter was sutured and fixed subcutaneously, and then, the outer section of the catheter was fixed between the ears after penetrating the skin. Finally, the paravertebral muscle and fascia were sutured, 10 μl of sterile saline was injected, and the catheter was sealed by melting the end. The rats were allowed to recover in individual cages for 3 days, and then, the drugs or vehicle were intrathecally administered: a microglial inhibitor MC (10 nmol/μl, 10 μl), an astrocytic inhibitor FC (1 nmol/μl, 10 μl), and an CXCR4-specific antagonist AMD3100 (1 μg/μl, 10 μl) (Yang et al., 2015, 2017a,b).

### Electron microscopy

After being anesthetized with sodium pentobarbital (50 mg/kg, i.p.), the rats were perfused with physiological saline, 2.5% glutaraldehyde (SPI Supplies, United States, SPI#02607-BA), and 4% paraformaldehyde (Sinopharm Chemical Reagent Co. Ltd., China) in 0.1 M PBS. The spinal cords were collected and fixed with 2.5% glutaraldehyde in 0.1 M PBS overnight at 4°C. The dorsal spinal cords were cut into 1 mm × 1 mm × 2 mm segments, prior to undergoing ossification in 1% OsO_4_ (TED PELLA Inc., United States, #18456) and dehydration with an ascending acetone series. The osmicated tissue blocks were further embedded in Epo812 (SPI Supplies, United States, SPI#02659-AB). The ultrathin sections (50 nm) were cut perpendicularly to the axis of the spinal cord with a diamond knife on an Ultratome (EM UC7, Leica, Germany) and collected by copper grids (200 meshes, 250 μm diameter for each mesh). The ultrathin sections stained with uranyl acetate and lead citrate were observed under an electron microscope (EM; Hitachi, TH7700, Tokyo, Japan), and microphotographs were captured by CCD camera using Hitachi TEM system (version: 01.06.06.37) at the same time. For details refer to Xie et al. (2013) For quantitative analysis, averaged number of normal (NC) or degenerative neuronal cell bodies (DC) per mesh (4.9 × 10^4^ μm) were counted from total 27 ultrathin sections of 9 copper grids obtained from the lumbar dorsal horn (*n* = 3 rats for both ITS and ITC); namely, three copper grids (3 × 200 meshes × 4.9 × 10^4^ μm) were prepared for each rat, and one copper grid contained three ultrathin sections.

### *In vivo* multi-electrode array recording

#### Surgery

The rat was anesthetized by isoflurane through gas anesthesia machine (R5101P, RWD, China) during the experiment session. The lumbar enlargement of the spinal cord (L3–L5 level) was exposed. The rat was attached to a stereotaxic apparatus (SR-6R, Narishige, Japan) by fixing the vertebrae with two vertebral clamps suspending the vertebral column in a horizontal direction. The dura matter and the pia matter of the spinal cord were carefully stripped by the 1-ml curved syringe needle. The bleeding was completely cleaned by vacuum pump (GL-802A, Kylin-Bell, China) under ACF perfusion. One end of the silver thread was placed into the spine for ground wire. Additionally, the hemostatic sponges absorbed 37°C ACF were used to cover the spinal cord for retaining moist and hemostasis during the surgery and recording.

#### Electrophysiological recording and data acquisition

As shown in our previous report (Wang et al., 2011), the electrophysiological signal of neurons from spinal cord was collected by two-stage amplifications in headstage and Pre-Amplifier (DigiAmp). A headstage (HST/32V-G20) was used to connect the silicon electrodes. The Pre-Amplifier consists of 32 channels through which analog-to-digital (A–D) conversion is processed and sent to the OmniPlex chassis. Ground wire of the silicon electrodes was connected to silver thread. Silicon electrodes of 32 channels (model: A4 × 2-tet-5 mm-150-200-121-OCM32LP, Neuronexus corporation, United States) had four feet in 200-μm interval. Each foot had two tetrodes with 150-μm interval. Each tetrode had four channels in 25-μm interval. Every channel site was square and its length of side was 11 μm (area: 121 μm^2^). The thickness of electrode was 15 μm. The OmniPlex chassis is the data processing center of the Plexon Multichannel acquisition processor (MAP) system and includes digital signal input from Pre-Amplifier. The MAP system is controlled by the two primary software components, namely, OmniPlex server and PlexControl. PlexNet and a software development kit (SDK) were used to conduct offline analysis. OmniPlex Server software receives data from hardware devices, ends commands to them, and contains the topology (network) of software modules, which perform filtering, thresholding, sorting, and other signal processing functions. PlexControl software is the main user interface to the MAP system and visualization of signals, spikes, and events, along with the control of all processing and recording parameters. The MAP system provides with simultaneous 40 KHz sampling rate through A-D conversion on each individual channel at a 12-bit resolution. The original signals in OmniPlex server software are separated into two primary components through digital signal processor (DSP) filters, namely, local field potentials (LFP) and the continuous spike signal (SPKC). LFP is lowpass filtering with a cutoff of approximately 200–300 Hz and then down sampled to a sampling rate of 1 KHz. SPKC is highpass filtering with a cutoff of approximately 200–300 Hz and then sampled at 40 KHz rate as the original signal. The MAP system in our laboratory contains two 16-channel SIG boards and one 32-channel DSP board that can support four digital input (DIs) and four digital output (DOs) sub-boards. National Instruments™ Data Acquisition (NI DAQ) devices (PCI-6071E, 1.25 M samples/s, 64 channels, 12-bit resolution) are used to support A–D subsystem plus accessories (C-HUB, 2 m CBL/C-HUB cable, and A/D board) for up to 64-channel analog recording.

**FIGURE 2:**
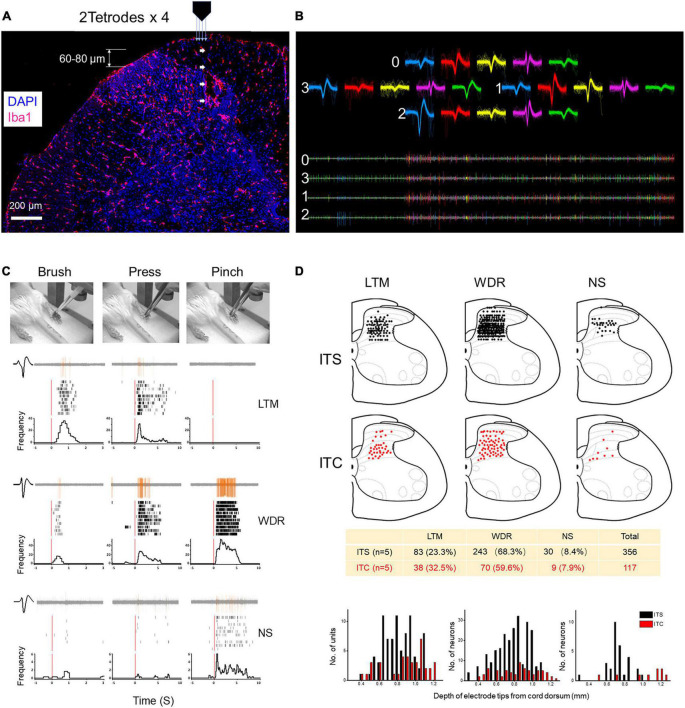
Extracellular MEA mapping of spinal dorsal horn neurons 7 days after ITS or ITC. **(A)** Photomicrograph showing tetrode track and MEA recording sites at the spinal dorsal horn labeled with Iba1 and DAPI. **(B)** Waveforms and spike traces of five units sorted out by one tetrode (0–3). **(C)** Identification of three classes of dorsal horn neurons (LTM, WDR, and NS) by electrophysiological properties to brush, pressure, and pinch stimuli applied to their corresponding peripheral receptive fields in one hind paw pad showing by peri-stimulus time histograms and raster displays. **(D)** Spatial localization and counts of three classes of units across the dorsal horn from the surface of the cord dorsum to the deep layers by 60- to 80-μm step advance following ITS (*n* = 5) and ITC (*n* = 5), respectively.

Single-unit spikes recorded from each channel were separated by principal component analysis (PCA) in real time and shown by a pair of time–voltage boxes per unit. The size and position of the time–voltage boxes are defined by the experimenter to isolate the waveforms that belonged to a given unit (Figures 2A,B). Spikes were only accepted as valid when they pass through both boxes (Wang et al., 2011). Raw and discriminated signals were fed through an audio monitor and were real time displayed by PlexControl software (Plexon, United States). Waveforms and recorded spike trains were stored on computer disk for offline analyses. Spike train analyses were performed using NeuroExplorer and MATLAB (MathWorks, Natick, MA, United States). Inter-spike interval (ISI) histograms of the continuous spike data could be obtained and used to reject the unit with the ISI plots that reveal counts in bins close to zero. Individual neurons were detected and clustered from raw data by KiloSort software (Marius et al., 2016), a freely available, automatic sorting, open-source tool. To view and manually curate the results of KiloSort, Phy software^1^ was used. During manual curation, every spike was identified based on the waveform consistency, amplitude (>50 μV), and PCA (Figure 2B).

#### Identification of dorsal horn neurons

In total, three classes of dorsal horn neurons were identified according to their response characteristics to natural mechanical stimuli (brush, pressure, and pinch) applied to their cutaneous receptive fields (You et al., 2003; Li and Chen, 2004; Li et al., 2008), namely, (1) class 1 (also known as low-threshold mechanoreceptive – LTM) neurons, mostly evoked by innocuous or non-painful stimulation; (2) class 2 (also known as multi-receptive or WDR) neurons, evoked by both noxious and non-noxious stimulation but with the response stronger to noxious stimulation; (3) class 3 (also known as nociceptive specific – NS) neurons, only evoked by noxious stimulation (Figure 2C). Among the three classes of neurons, spontaneous type and non-spontaneous type were also identified according to the number of spikes of basal activity at the initial 5-min recording. Non-spontaneous type was defined as if the number of spikes was less than 10 at the initial 5-min recording, otherwise was spontaneous type.

### Fluorescent immunohistochemistry

**FIGURE 3:**
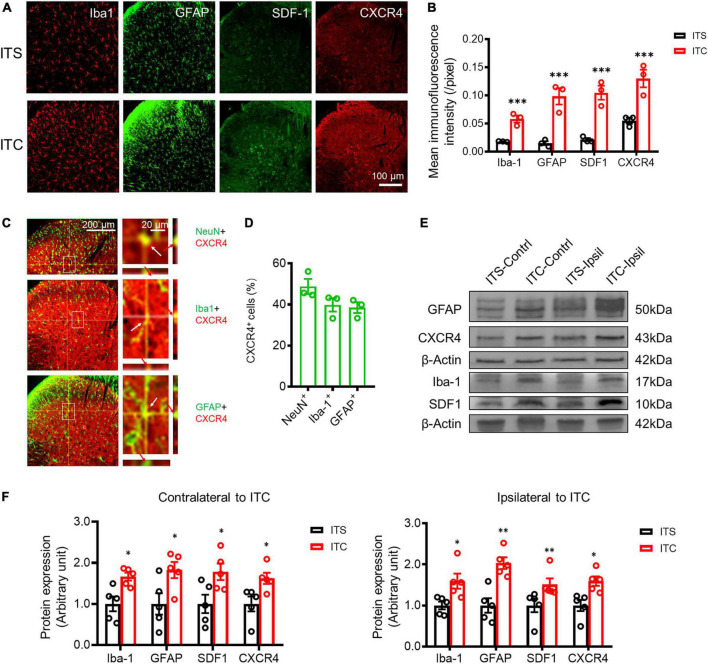
Secondary neuroinflammation in the spinal dorsal horn 7 days after ITS or ITC. **(A)** Representative immunofluorescent photomicrographs showing the expression of Iba-1, GFAP, SDF1, and CXCR4 at the spinal dorsal horn contralateral to ITS or ITC. **(B)** Quantification of the mean immunofluorescent intensity of Iba-1, GFAP, SDF1, and CXCR4-labeled cellular profiles. ***p < 0.001 ITC vs. ITS, *n* = 3 rats/group. **(C)** Representative immunofluorescent photomicrographs showing co-localization of CXCR4 with NeuN, Iba-1, and GFAP-labeled cellular profiles at the spinal dorsal horn contralateral to ITC by orthogonal viewing method. **(D)** The percentage of CXCR4 in NeuN, Iba-1, and GFAP-labeled cellular profiles. **(E)** Western blot assay of Iba-1, GFAP, SDF1, and CXCR4 in bilateral dorsal spinal cord 7 days after ITS or ITC. **(F)** Quantitative analysis of the Western blot results for Iba-1, GFAP, SDF1, and CXCR4 in bilateral dorsal cord following 7 days after ITS or ITC. *p < 0.05, **p < 0.01, ITC vs. ITS, *n* = 5 rats/group.

As in our previous report (Yang et al., 2015, 2016, 2017a,b), the rats were anesthetized with sodium pentobarbital (50 mg/kg, i.p.), then perfused with physiological saline, and followed by 4% paraformaldehyde in 0.1 M PBS solution. The spinal cord was removed and postfixed overnight at 4°C and then immersed in 30% sucrose in 0.1 M PBS. Spinal tissues were cut into transverse sections (25 μm thick) on CM1900 freezing microtome (Leica, Germany). After blocking with 2% BSA in 0.1 M PBS, the sections were incubated overnight at 4°C with the primary antibodies: mouse anti-NeuN (1:200, Abcam), rabbit anti-Bax (1:200, Abcam), rabbit anti-Bcl-2 (1:200, Abcam), goat anti-Iba1(1:200, Abcam), mouse anti-GFAP(1:200, Millipore), mouse anti-SDF1 (1:200, Santa), and rabbit anti-CXCR4 (1:400, Alomone) (for details refer to Supplementary Table 1). On the following day, Cy3- or FITC-conjugated secondary antibodies were incubated for 3 h at room temperature. For double immunostaining, sections were incubated with a mixture of primary antibodies overnight at 4°C, followed by a mixture of secondary antibodies. The images were examined under a laser scan confocal fluorescent microscope (Olympus FV1000, Japan) and captured at 1024 × 1024 resolution pixels. The fluorescent intensity of cellular profiles in the spinal dorsal horn was analyzed by ImageJ software (Java 1.6.024). There were three rats in each group, and at least 10 immunofluorescent photomicrographs were taken and analyzed in this study. The co-localization of two immunofluorescent products was analyzed with an orthogonal view (Figure 3C), and NeuN + CXCR4-positive, GFAP + CXCR4-positive, and Iba1 + CXCR4-positive cellular profiles were counted and analyzed from three rats, respectively.

### Fluorescent double-labeling method of TdT-mediated dUTP nick end labeling and 2-(4-amidinophenyl)-6-indolecarbamidine dihydrochloride

The sections of the spinal cord described above were washed three times by PBS, and the nuclei were counterstained with 4′,6-diamidino-2-phenylindole (DAPI, 1 g/ml, Sigma) for 5 min. After being washed with PBS, the sections were incubated in permeabilization solution (0.1% Triton X-100, 0.1% sodium citrate, freshly prepared) for 15 min on ice. According to the instructions of the TUNEL (terminal deoxynucleotidyl transferase-mediated nick end labeling) kit (Roche, Switzerland, Cat# 684 795 910), proper amount of detection solution was added sequentially and washed three times after incubation at 37°C for 1 h.

### Western blotting

As described previously (Yang et al., 2015, 2016, 2017a,b), the lumbar spinal cord (L3–L5) was obtained from each rat under deep anesthesia with sodium pentobarbital (50 mg/kg, i.p.) after behavioral testing. The tissues were homogenized in a mixture of protease inhibitors and radioimmunoprecipitation assay (RIPA) lysis buffer. Total proteins were extracted by centrifugation at 12,000 *g* for 10 min at 4°C, and protein concentrations were determined using a BCA Protein Assay kit. The samples containing same amounts of protein were heated for 10 min at 95°C, separated on sodium dodecyl sulfate-polyacrylamide gel electrophoresis (SDS-PAGE) gels by electrophoresis (Bio-Rad), and transferred onto PVDF membranes. The membranes were blocked with 5% skim milk for 3 h at room temperature and incubated with anti-β-actin and primary antibodies mentioned above (for details refer to Supplementary Table 1) overnight at 4°C and then with horseradish peroxidase-labeled secondary antibodies at room temperature for 2 h. The membranes were displayed with enhanced chemiluminescence reagents and images captured using Fluor Chem FC2 (Alpha Innotech Corp.).

### Statistical analysis

All data were expressed as mean ± SEM. Differences in changes of values of each group were tested using non-parametric *U*-test, *t*-test, and one-way ANOVA, followed by individual Bonferroni *post hoc* test. The behavioral data collected over time among the groups were compared by two-way repeated-measures ANOVA followed by Bonferroni *post hoc* tests. A level of *p* < 0.05 was accepted as significant (for details, refer to Supplementary Tables 1, 2).

## Results

### Secondary neuronal death occurs in spinal dorsal horn caused by experimental thalamic hemorrhagic stroke

Similar to our previous reports (Yang et al., 2014, 2016, 2017a,b), unilateral ITC injection confined to the VBC/Po resulted in bilateral reductions in the PWMT (Figures 1A,B), suggesting the occurrence of steady bilateral mechanical pain hypersensitivity after experimental THS. Compared with ITS control, rats with THS had lowered PWMT by 50% on 3rd day after ITC, whereas the values were bottomed on 7th day after ITC (*p* < 0.001, ITC-Ipsil vs. ITS-Ipsil; *p* < 0.001, ITC-Contrl vs. ITS-Contrl; *p* < 0.001, ITC -1 day vs. 3 day, ITC -1 day vs. 7 day, ITC -1 day vs. 10 day, ITC 3 day vs. 7 day, ITC 3 day vs. 10 day) (Figure 1B and Supplementary Table 2).

**FIGURE 4:**
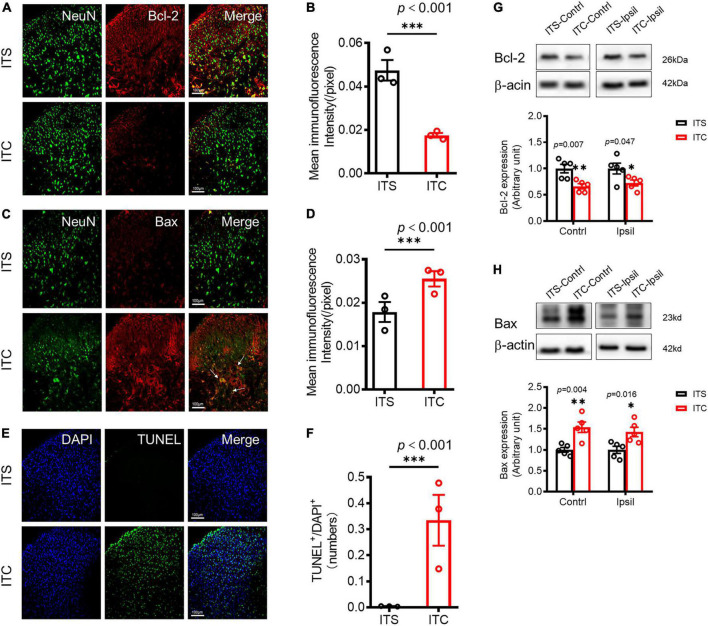
Retrograde degeneration induced neuronal cell apoptosis of spinal dorsal horn caused by ITC-induced thalamic hemorrhagic stroke. Representative immunofluorescent photomicrographs showing co-localization of NeuN with Bcl-2 **(A)** and with Bax **(C)** in the spinal dorsal horn 7 days after ITC. Quantification of the mean immunofluorescent intensity of Bcl-2 **(B)** and Bax **(D)**. ***p < 0.001 ITC vs. ITS, *n* = 3 rats/group. **(E)** Representative fluorescent photomicrographs of TUNEL- and DAPI-labeled cells. **(F)** Quantitative data of TUNEL-labeled cells. ***p < 0.001 ITC vs. ITS, *n* = 3 rats/group. **(G)** and **(H)** Western blot assay and quantitative analysis of Bcl-2 **(G)** and Bax **(H)** protein expressions in bilateral dorsal cord following 7 days after ITC or ITS. *p < 0.05, **p < 0.01, ITC vs. ITS, *n* = 5 rats/group.

To examine whether there is secondary damage of the STT projecting neuronal cell bodies in the spinal dorsal horn (Figure 1C), the expression of apoptosis factors was first quantified by immunohistochemistry, and then, the apoptotic cells were detected by TUNEL staining (Matsushita et al., 2000). Apoptosis-inhibiting molecule Bcl-2/NeuN co-labeled neuronal profiles were decreased (*p* < 0.001, ITC vs. ITS) (Figures 4A,B and Supplementary Table 2), whereas apoptosis-promoting molecule Bax/NeuN co-labeled neuronal profiles were dramatically increased in the spinal dorsal horn 7 days after ITC (*p* < 0.001, ITC vs. ITS) (Figures 4C,D and Supplementary Table 2), relative to ITS, respectively (Figures 4A–D). The TUNEL-labeled cell profiles were also significantly increased in the spinal dorsal horn 7 days after ITC relative to ITS (*p* < 0.001, ITC vs. ITS) (Figures 4E,F and Supplementary Table 2). Surprisingly interesting, the TUNEL-labeled cell profiles could be seen in both sides of the spinal dorsal horn although the side contralateral to ITC was predominant (Supplementary Figure 1A). Moreover, a few TUNEL-labeled cell profiles were also seen in the ventral horn of the spinal cord (Supplementary Figure 1A). The decreased expression of Bcl-2 and increased expression of Bax proteins in bilateral dorsal horn 7 days after ITC, relative to ITS, were reconfirmed by Western blot technique (Figures 4G,H, Supplementary Tables 2, 3).

**FIGURE 5:**
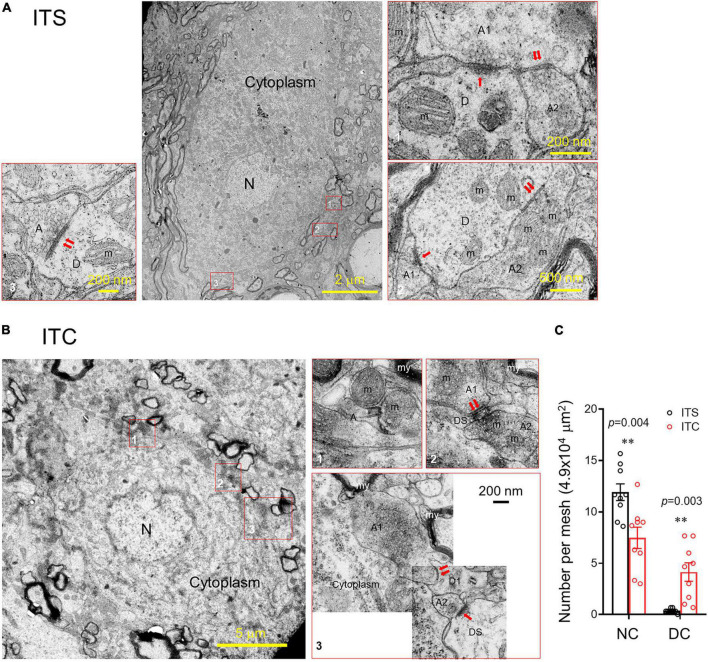
Ultrastructural evidence for retrograde degeneration of spinal dorsal horn neuronal cell bodies and altered synaptic structures. **(A)** A normal neuronal cell body and adjacent synaptic structures 7 days after ITS. **(B)** A degenerating neuronal cell bodies with altered nucleus, damaged cell membranous structures and mitochondrial swelling, vesiculation, and disappearance of the crista and altered synaptic structures 7 days after ITC. A, axon; D, dendrite; DS, dendritic spine; m, mitochondrion; my, myelin; N, nucleus; ↑asymmetric synapse;↑↑symmetric synapses. **(C)** Averaged number of normal neuronal cell bodies (NC) and degenerative neuronal cell bodies (DC) from the spinal dorsal horn following 7 days after ITC or ITS. **p < 0.01, ITC vs. ITS, *n* = 3 rats/group, with nine ultrathin sections being observed and counted from each rat.

Ultrastructural observation under EM identified distinct neuronal degeneration and altered synaptic structures in the spinal dorsal horn 7 days after ITC, whereas they were less seen following ITS (Figure 5). In sharp contrast to the normal neuronal cell body (Figure 5A), the degenerative cell body had shrinkage of nucleus and perikaryon accompanied by the disruption of membranous system and loss of organelles (mitochondria, Golgi’s apparatus, endoplasmic reticulum, etc.) (Figure 5B). Quantitative analysis showed decreased number of normal cell bodies and increased number of degenerative cell bodies in rats with ITC relative to ITS (Figure 5C; Supplementary Table 2). Meanwhile, the synaptic structures (axon–dendrite, axon-dendritic spine, or axon–axon) adjacent to the degenerative cell body were also significantly tortured in morphology in both presynaptic and post-synaptic components (right panel of Figure 5B). For example, comparing to the normal synapses (left and right panels of Figure 5A), normal mitochondria were less seen and they were replaced by ones with swelling and disrupted mitochondrial cristae in both pre- and post-synaptic structures. The vesicles were less seen at the presynaptic bank of the post-synaptic active zones, and instead, they were abnormally accumulated far away from it.

To map the spinal dorsal horn neuronal activities, extracellular MEA (2 tetrode × 4 = 32 channels) recordings were performed at the contralateral spinal dorsal horn of anesthetized rats with ITS (*n* = 5) or ITC (*n* = 5). As aforementioned in the methodology, neuronal activities were recorded and identified by applying mechanical stimuli to the peripheral receptive fields at each step toward the deep of the dorsal horn from cord dorsum by advancing the MEA electrodes every 60–80 μm (total 600 to 800 μm in depth). The results revealed significant loss of neurons of all three classes in terms of LTM (ITC vs. ITS: 37 vs. 83 units), WDR (ITC vs. ITS: 68 vs. 243 units), and NS (ITC vs. ITS: 9 vs. 30 units) across from superficial to deep layers (Figure 2D).

The above structural and functional results demonstrated that the THS-induced damage of primary STT projecting fiber terminals in the stroke locus could result in STT projecting neuronal cell apoptosis and death at the spinal dorsal horn through axonal retrograde degeneration process as we proposed (Figure 1C).

### Secondary neuroinflammation in the spinal dorsal horn caused by experimental thalamic hemorrhagic stroke

To examine whether there is secondary neuroinflammation in the spinal dorsal horn following the THS-induced axonal retrograde degeneration, activations of microglial and astrocytic cells that have been demonstrated to play the key roles in the mediation of neuroinflammation (Timotijević et al., 2012) were detected by both immunofluorescence and Western blot techniques (Yang et al., 2015, 2017a,b). Comparing to the ITS, the cellular profiles labeled by Iba-1 (a microglial biomarker) and GFAP (an astrocytic biomarker) were both significantly increased in the spinal dorsal horn following 7 days after ITC (*p* < 0.001, ITC vs. ITS), respectively (Figures 3A,B; Supplementary Table 2), suggesting the occurrence of neuroinflammation. SDF1 and its receptor CXCR4 have been shown to play the critical roles in the mediation of neuroinflammation (Yang et al., 2015, 2017a,b), and the expressions of both proteins were also examined. The cellular profiles labeled by SDF1 and CXCR4 were also significantly increased in the spinal dorsal horn 7 days after ITC, relative to ITS (*p* < 0.001, ITC vs. ITS) (Figures 3A,B; Supplementary Table 2). To define the cellular distribution of CXCR4, double labeling of CXCR4 with different cell markers was performed and showed that CXCR4-labeled profiles were co-localized with about 40–50% of NeuN-, Iba- 1-, and GFAP-labeled profiles in the spinal dorsal horn (*n* = 3 rats) (Figures 3C,D; Supplementary Table 2). Of total 299 NeuN^+^ (99.67 ± 0.88 per rat), 146 was CXCR4^+^/NeuN^+^ (48.67 ± 3.18 per rat), whereas of total 287 Iba1^+^ (95.67 ± 6.64 per rat) and 311 GFAP^+^ (103.67.00 ± 3.28 per rat), 115 was CXCR4^+^/Iba1^+^ (38.33 ± 5.36 per rat) and 120 was CXCR4^+^/GFAP^+^ (40.00 ± 3.06 per rat), suggesting that CXCR4 can be co-expressed in both neuronal cells and the two types of glial cellular profiles. Western blot assays also showed significant increase in protein expression levels of both Iba-1 (*p* = 0.011, ITC-Contrl vs. ITS-Contrl; *p* = 0.021, ITC-Ipsil vs. ITS-Ipsil) and GFAP (*p* = 0.035, ITC-Contrl vs. ITS-Contrl; *p* = 0.002, ITC-Ipsil vs. ITS-Ipsil) and SDF1 (*p* = 0.009, ITC-Contrl vs. ITS-Contrl; *p* = 0.032, ITC-Ipsil vs. ITS-Ipsil) and CXCR4 (*p* = 0.025, ITC-Contrl vs. ITS-Contrl; *p* = 0.010, ITC-Ipsil vs. ITS-Ipsil) in both sides of the dorsal spinal cord (Figures 3E,F, Supplementary Tables 2, 3). These results suggest the occurrence of secondary neuroinflammation involving SDF1-CXCR4 signaling in the spinal dorsal horn caused by primary THS *via* mechanisms of axonal retrograde degeneration of the STT projecting neuronal cells. Moreover, Iba1- and GFAP-labeled cellular profiles were also seen in the spinal ventral horn, and the immunofluorescent intensity of these two proteins was significantly increased in the ITC group relative to ITS control (Supplementary Figures 1B,C), suggesting the occurrence of a weak secondary neuroinflammation in the spinal ventral horn in response to some ventral horn neuronal death (Supplementary Figure 1A).

### Central sensitization of the spinal dorsal horn neurons caused by primary thalamic hemorrhagic stroke

**FIGURE 6:**
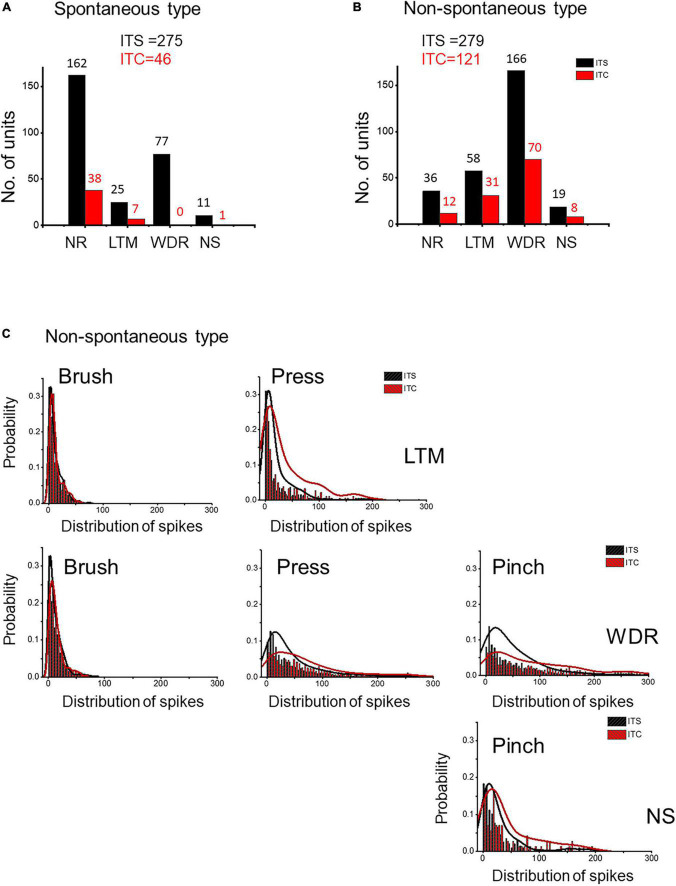
Evidence for neuronal hyperexcitability or sensitization in spared spinal dorsal horn neurons 7 days after ITS or ITC. MEA recording and analysis of spontaneous type **(A)** and non-spontaneous type **(B)** of the three classes of spinal dorsal horn units following ITS and ITC. **(C)** Probability of frequency distribution of spike responses in LTM, WDR, and NS units to brush, pressure, and pinch stimuli in the spared non-spontaneous type following ITS or ITC. The frequency of spike responses of all three classes of dorsal horn neurons is stimulus intensity-dependent, and ITC group shows more high-frequency spike discharges than ITS group when stimulated by high-intensity mechanical stimuli (pressure and pinch) to both WDR and NS as well as LTM.

According to basal activities in the dorsal horn neurons, extracellular MEA recordings identified two types of spinal units: spontaneous type with basal spike activities (ITC vs. ITS: 46 vs. 275 units) and non-spontaneous type without basal spike activities (ITC vs. ITS: 121 vs. 279 units) (Figures 6A,B). Following 7 days after ITC, few responding WDR (*n* = 0) and NS (*n* = 1) units were recorded for the spontaneous type, while many responding WDR (*n* = 70) and NS (*n* = 8) units were recorded for the non-spontaneous type, indicating that majority of spontaneous type but not non-spontaneous type of WDR and NS units are thalamic projecting STT neurons (Figures 6A,B). There was also great loss of non-responding (NR) units from both spontaneous type (76%) and non-spontaneous type (66%) (Figures 6A,B).

In the remaining non-spontaneous type of three classes of neurons recorded 7 days following ITC, high-intensity mechanical stimuli (pressure and pinch) induced more spike discharges in both WDR and NS and LTM units compared with ITS group, whereas no significant change could be seen when low-intensity mechanical stimulus (brush) was applied to the peripheral receptive fields of LTM and WDR neurons between ITC and ITS (Figure 6C). These results suggest that there is central sensitization in three classes of the spinal dorsal horn neurons in the surviving non-spontaneous type under secondary spinal neuronal damage-induced neuroinflammation.

### Relief of thalamic hemorrhagic stroke pain hypersensitivity by intrathecal pharmacological suppression of secondary spinal neuroinflammation

**FIGURE 7:**
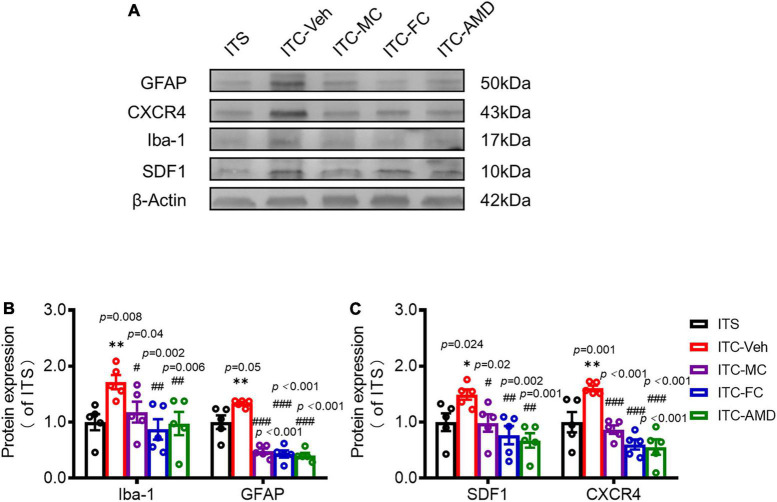
Suppressing effects of i.t. antineuroinflammation treatment on expression levels of Iba-1, GFAP, SDF1, and CXCR4 in the spinal dorsal horn following ITS or ITC. Western blot assays **(A)** and quantitative analysis **(B–C)** of Iba1, GFAP, SDF-1, and CXCR4 expressions in the dorsal cord following i.t. administration of vehicle and drugs 10 days after ITS or ITC (3 days after i.t. catheterization). ITC-Veh, rats with ITC receiving i.t. administration of vehicle; ITC-MC, rats with ITC receiving i.t. administration of MC; ITC-FC, rats with ITC receiving i.t. administration of FC; ITC-AMD, rats with ITC receiving i.t. administration of AMD3100. *p < 0.05, **p < 0.01, ITS vs. ITC-Veh; #*p* < 0.05, ##*p* < 0.01, ###*p* < 0.001, ITC-MC/ITC-FC/ITC-AMD vs. ITC-Veh; *n* = 5 rats/group.

**FIGURE 8:**
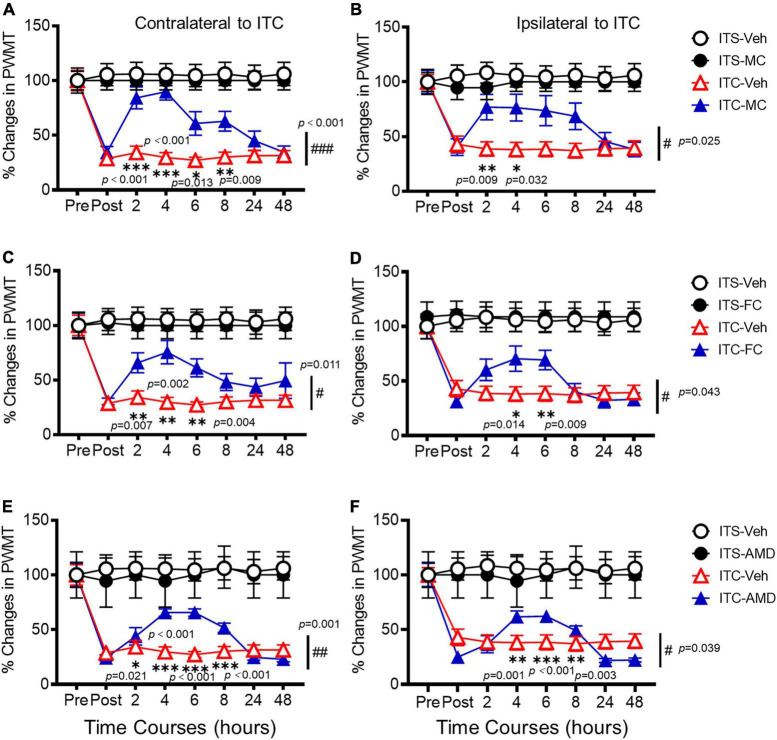
Analgesic effects of i.t. antineuroinflammation treatment on thalamic hemorrhagic stroke pain hypersensitivity. Time courses of the analgesic effects following i.t. administration of MC **(A,B)**, FC **(C,D)**, and AMD3100 (AMD) **(E,F)** on the THS mechanical pain hypersensitivity 10 days after ITS or ITC (3 days after i.t. catheterization). Pre, the day before ITS or ITC; Post 2, 4, 6, 8, 24, and 48 h, time after i.t. treatment of vehicle or drug on day 10 after ITS or ITC (or day 3 after i.t. catheterization). Abbreviations see Figure 7. *n* = 12 rats for ITS-Veh, ITC-Veh; *n* = 5 rats for ITS-MC; *n* = 11 rats for ITC-MC; *n* = 6 rats for ITS-FC; *n* = 10 rats for ITC-FC; *n* = 5 rats for ITS-AMD; *n* = 10 rats for ITC-AMD. *p < 0.05, **p < 0.01,; #*p* < 0.05, ##*p* < 0.01, ###*p* < 0.001, ITC-MC/ITC-FC/ITC-AMD vs. ITC-Veh.

To determine whether SDF1-CXCR4 signaling-mediated neuroinflammation contributes to the persistence of the THS pain hypersensitivity, we finally examined the pharmacological effects of i.t. administration of microglia inhibitor (MC), astrocytic inhibitor (FC), and CXCR4 antagonist (AMD3100, AMD) on the protein expression levels of Iba-1, GFAP, SDF1, and CXCR4 using Western blot and the THS mechanical pain hypersensitivity using behavioral assays 10 days after ITC or ITS (3 days after i.t. catheterization). The results showed that i.t. administration of MC, FC, and AMD, respectively, could block the THS-induced increase in expression levels of spinal Iba1, GFAP, SFD1, and CXCR4, relative to vehicle (Figure 7A). Quantitative analysis showed statistically significant decrease in expression levels of all four proteins in bilateral dorsal cord following i.t. antineuroinflammatory treatments by MC, FC, and AMD, respectively, relative to vehicle (Iba-1: *p* = 0.008, ITC-Veh vs. ITS; *p* = 0.040, ITC-MC vs. ITC-Veh; *p* = 0.002, ITC-FC vs. ITC-Veh; *p* = 0.006, ITC-AMD vs. ITC-Veh; GFAP: *p* = 0.005, ITC-Veh vs. ITS; *p* < 0.001, ITC-MC vs. ITC-Veh; *p* < 0.001, ITC-FC vs. ITC-Veh; *p* < 0.001, ITC-AMD vs. ITC-Veh; SDF1: *p* = 0.024, ITC-Veh vs. ITS; *p* = 0.020, ITC-MC vs. ITC-Veh; *p* = 0.020, ITC-FC vs. ITC-Veh; *p* = 0.001, ITC-AMD vs. ITC-Veh; and CXCR4: *p* = 0.001, ITC-Veh vs. ITS; *p* < 0.001, ITC-MC vs. ITC-Veh; *p* < 0.001, ITC-FC vs. ITC-Veh; *p* < 0.001, ITC-AMD vs. ITC-Veh) (Figures 7B,C, Supplementary Tables 2, 3). The same treatment also significantly ameliorated the THS-induced bilateral mechanical pain hypersensitivity (*p* < 0.001, ITC-MC vs. ITC-Veh Contralateral; *p* = 0.025, ITC-MC vs. ITC-Veh Ipsilateral; *p* = 0.011, ITC-FC vs. ITC-Veh Contralateral; *p* = 0.043, ITC-FC vs. ITC-Veh Ipsilateral; *p* = 0.001, ITC-AMD vs. ITC-Veh Contralateral; *p* = 0.039, ITC-AMD vs. ITC-Veh Ipsilateral) (Figure 8, Supplementary Tables 2, 3). However, the same i.t. treatment did not change the basal mechanical pain sensitivity relative to the ITS control (*p* = 0.516, ITS-MC vs. ITS-Veh Contralateral; *p* = 0.589, ITS-MC vs. ITS-Veh Ipsilateral; *p* = 0.551, ITS-FC vs. ITS-Veh Contralateral; *p* = 0.521, ITS-FC vs. ITS-Veh Ipsilateral; *p* = 0.909, ITS-AMD vs. ITS-Veh Contralateral; *p* = 0.945, ITS-AMD vs. ITS-Veh Ipsilateral) (Figure 8, Supplementary Tables 2, 3). All these results suggest that both microglia and astrocytes and SDF1-CXCR4 signaling in the spinal cord dorsal horn play the important roles in maintaining the THS-induced mechanical pain hypersensitivity.

## Discussion

The major gain of this study in understanding of the underlying mechanisms of CPSP is as follows: (1) in addition to the thalamic pathological changes *per se* (Wasserman and Koeberle, 2009; Yang et al., 2014, 2016, 2017a; Cai et al., 2018; Huang et al., 2020; Fu et al., 2021), the unilateral experimental THS can also result in secondary neuronal death and neuroinflammation in bilateral spinal dorsal horn demonstrated by both structural and functional techniques; (2) although other factors, such as Fgr-NFκB-ERK1/2 signaling, fat-mass and obesity-associated protein (FTO)-TLR4 signaling, and PSD95-nNOS linking, have been shown to be involved in the processing of CPSP at the thalamic level (Cai et al., 2018; Huang et al., 2020; Fu et al., 2021), SDF1-CXCR4 signaling is likely to play the critical roles in the mediation of neuroinflammation *via* glial–glial and glial–neuronal interactions at both primary thalamic injury site (Yang et al., 2014, 2016, 2017a) and secondary injury site at the spinal dorsal horn *via* retrograde degeneration; (3) spared spinal dorsal horn neuronal hyperexcitability or central sensitization can be produced by secondary neuronal damage-induced neuroinflammation that may contribute to the maintenance of THS mechanical pain hypersensitivity; (4) pharmacological effectiveness in relief of experimental THS mechanical pain hypersensitivity through i.t. route of antineuroinflammation provides a proof-of-concept evidence for clinical therapeutic approach targeting on primary sensory input in patients with CPSP (Haroutounian et al., 2018; Choi et al., 2021b). Collectively, our study provided a new line of experimental evidence demonstrating that secondary neuronal death and neuroinflammation in the spinal dorsal horn can be induced by primary thalamic damage *via* axonal retrograde degeneration process and the spinal dorsal horn might be an important site for the treatment of CPSP in clinic.

### Primary thalamic hemorrhagic stroke results in spinal dorsal horn neuronal cell death through retrograde degeneration process

Nociceptive withdrawal reflex is known to be mediated by a spinally organized neural circuitry, which mainly includes a central processing site of sensory information in the dorsal horn and a central processing site for motor output in the ventral horn (You et al., 2003; Li and Chen, 2004; Li et al., 2008). This reflex is a useful and powerful readout for the evaluation of pain (nociceptive) responses. However, how thalamic hemorrhagic damage causes bilateral mechanical pain hypersensitivity is not fully understood. In our previous reports (Yang et al., 2014, 2016, 2017a), we have examined the THS effect on thermal and mechanical pain sensitivity using both radiant heat and von Frey filament. The results show that in the rat model, unilateral ITC results in bilateral mechanical pain hypersensitivity but without thermal pain hypersensitivity that is likely to be different from what has been seen in the mouse model (Yang et al., 2014; Cai et al., 2018; Huang et al., 2020; Fu et al., 2021). We still do not know exactly why there is species difference in pain response between rats and mice even using similar strategy of ITC targeting on the similar region at the thalamus. However, when looking carefully at the THS site, the rat model involves an area including VBC/Po/ML (Yang et al., 2014), whereas the mouse model only involves VBC (VPM/VPL), suggesting that the THS size may determine the characteristics of pain response due to the damage of different components of somatosensory system in the CNS. Because it has been demonstrated that intra-thalamic microinjection of SDF1 into VBC/Po/ML can produce bilateral mechanical pain hypersensitivity as well (Yang et al., 2017a), and intra-thalamic microinjections and i.t. administration of the same antineuroinflammatory drugs (MC, FC, and AMD) are also effective to ameliorate bilateral mechanical pain hypersensitivity induced by THS in rats (Yang et al., 2017a and the current result), the unilateral THS-induced bilateral mechanical pain hypersensitivity should be mediated by both thalamus and spinal cord.

Our present study found that unilateral primary THS-induced neural damage could result in secondary neuronal death in bilateral spinal dorsal horn, suggesting the occurrence of STT projecting neuronal cell death *via* axonal retrograde degeneration. Although secondary neuronal damage was detected in both sides of dorsal horn following unilateral primary THS, predominance was seen in the contralateral side to the ITC (Supplementary Figure 1A). This result suggests that STT neurons may send bilateral projections to the thalamus in rats. Moreover, besides dorsal horn, a few TUNEL-labeled cellular profiles were also seen in the ventral horn of the spinal cord (Supplementary Figure 1A). Because there is no direct projection from the spinal ventral horn to the thalamus, the apoptotic ventral horn cells may be caused by transsynaptic degeneration along the noxious flexor reflex circuitry from dorsal to ventral horn. It was also noted that some TUNEL-labeled cellular profiles existed in the ventral and lateral columns of white matter in the spinal cord (refer to Supplementary Figure 1A). Although we did not get insight into the details, apoptosis of mature myelinating oligodendrocytes and oligodendrocyte progenitor cells might occur due to myelin damage of the STT fibers in the ventral column *via* retrograde axonal degeneration following the unilateral THS (Chen and Guan, 2017). The TUNEL-labeled cellular profiles seen in the lateral column (refer to Supplementary Figure 1A) may be caused by orthograde degeneration of the corticospinal tract (CST) fibers passing through the internal capsule (IC) affected by unilateral THS. If any, the apoptotic response seen in the ventral horn may also be caused by the IC damage involving the CST fibers. However, case analysis showed that single damage to the IC by ITC did not result in any pain hypersensitivity (Yang et al., 2014), excluding the role of the lateral column changes in the processing of CPSP. Although strong evidence is still lacking due to lower resolution of the macroscopic brain imaging techniques, some human case DTT studies showed abnormal integrity of STT in patients with CPSP (Jang et al., 2019; Park et al., 2021), highly supporting our current observations from mesoscopic to microscopic approaches. Taken all these into account, we concluded that primary THS results in secondary neuronal death in the spinal dorsal horn *via* axonal retrograde degeneration.

### Secondary spinal dorsal horn neuroinflammation caused by primary thalamic hemorrhagic stroke through retrograde degeneration

Following stroke, the primary lesion may disrupt functional connections in adjacent and remotely connected areas, resulting in the development of secondary injuries characterized with multiple pathological processes (Kluge et al., 2018; Cao et al., 2020, 2021; Necula et al., 2022; Park et al., 2022). The secondary neuroinflammation is a well-known pathological phenomenon, which is featured by the activation of glial cells at regions far from the primary stroke lesion due to retrograde or anterograde degeneration (Cao et al., 2020, 2021). We here showed evidence of substantial activation of microglia and astrocytes in the distant lumbar spinal dorsal horn after THS, suggesting the existence of secondary spinal neuroinflammation. The production of chemokines as a part of an inflammatory cascade is also implicated in the process of secondary neuroinflammation. In a model of spinal cord injury, CCL21 was considerably upregulated and involved in the mediation of the secondary microglial activation in the thalamus (Zhao et al., 2007). In parallel, we found that another classical pro-inflammatory chemokine, SDF1, and its cognate receptor CXCR4 were drastically increased in the spinal cord after THS, providing additional evidence for the development of secondary neuroinflammatory responses. Additionally, we demonstrated that CXCR4 is abundantly expressed in neurons, microglia, and astrocytes in the dorsal horn of the spinal cord, which is consistent with previous studies (Bai et al., 2016; Li et al., 2016). Although we did not investigate the cellular localization of SDF1 in this experiment, the literature indicates that activated microglia and astrocytes are its main sources (Shen et al., 2014; Bai et al., 2016; Hang et al., 2016; Yang et al., 2017a). Our pharmacological results also proved that microglia and astrocytes are the sources of SDF1 since i.t. treatment of microglial and astrocytic inhibitors was able to reverse the increase of SDF1 in the spinal cord. Signaling between SDF1 and CXCR4 in glial networks has been shown to be critical for triggering and maintaining neuroinflammation by mediating microglial–astrocytic interactions (Shen et al., 2014; Yang et al., 2017a). Notably, i.t. administration of the CXCR4 antagonist AMD3100 decreased the upregulation of SDF1 and CXCR4 as well as the activation of spinal microglia and astrocytes following THS, indicating that SDF1-CXCR4 signaling might mediate the secondary spinal neuroinflammation through a positive feedback mechanism as what we have observed in the THS region (Yang et al., 2017a).

### Spinal central sensitization caused by stromal cell-derived factor 1-CXCR4 mediated secondary neuroinflammation contributes to thalamic hemorrhagic stroke-induced pain hypersensitivity

Central sensitization, commonly described as the increased responsiveness of nociceptive neurons in the CNS to their normal or subthreshold afferent input, has been shown to underlie both heat and mechanical pain hypersensitivity (hyperalgesia and allodynia) in a variety of pathological situations (You et al., 2003; Li and Chen, 2004; Li et al., 2008; Vardeh et al., 2016; Ji et al., 2018). Experimental and clinical studies have shown that the enhanced thalamic electrical activity is involved in the development of CPSP (Kuan et al., 2015; Hirato et al., 2021). However, the existence of spinal sensitization in CPSP has not been shown. Our present study addressed this issue. The evidence for the presence of spinal sensitization stems from the followings: (i) the spared WDR, NS, and LTM neurons revealed by MEA recordings were more excitable under the THS condition; (ii) the capability of non-painful mechanical to elicit increased spinally organized nociceptive withdrawal reflex in the THS rats. In support, our previous study confirmed that the α2σ-1 subunit of the voltage-gated Ca^2+^ channel, which facilitates neuronal excitability, was upregulated in bilateral spinal dorsal horn after THS (Yang et al., 2016). Moreover, our present work also showed that i.t. antineuroinflammatory therapy, by inhibiting spinal activations of microglia and astrocytes, respectively, or by antagonizing against CXCR4, was able to dramatically reduce THS-induced mechanical pain hypersensitivity, suggesting that SDF1-CXCR4 signaling-mediated neuroinflammation results in spinal sensitization and contributes to CPSP. Indeed, a growing body of evidence has demonstrated that SDF1-CXCR4 signaling plays a crucial role in pathological pain (Luo et al., 2016a,b). The constitutive location of CXCR4 in neuronal and glial cells together with the inducible expression pattern and release of its endogenous ligand SDF1 from glial cells under pathological states enables SDF1-CXCR4 signaling to mediate pain by regulating glial-neuronal crosstalk (Liu et al., 2019; Hu et al., 2020; Cheng et al., 2021). This assumption is corroborated by previous studies demonstrating that SDF1-CXCR4 signaling could evoke neuronal hyperexcitability *via* enhancing the expression and function of Nav1.8 and Nav1.9 (Yang et al., 2015; Qiu et al., 2016). In the rat THS model, intra-thalamic injection of CXCR4 antagonist or glial inhibitor was sufficient to reduce neuronal and glial activation as well as mechanical pain hypersensitivity, further supporting that neuroinflammation mediated by SDF1-CXCR4 signaling is associated with pain (Yang et al., 2017a). SDF1-CXCR4 signaling in the spinal cord dorsal horn also contributes to the development and maintenance of pain directly by causing neuronal hyperexcitability or indirectly by enhancing neuronal excitability through facilitating glial-glial and glial-neuronal crosstalk, promoting releases of pro-inflammatory mediators (IL1β, IL6, and TNFα), and eventually boosting central sensitization (Shen et al., 2014; Luo et al., 2016a,b; Hu et al., 2017; Xing et al., 2017; Xu et al., 2017; Li et al., 2019; Liu et al., 2019). Our current data complemented these studies and verified that blocking SDF1-CXCR4 signaling-mediated neuroinflammation in the spinal cord was effective to ameliorate the TSH-induced pain hypersensitivity. In addition to what happened in the dorsal horn, we, in this study, also observed weak secondary neuronal death and neuroinflammation in the ventral horn of the spinal cord. Given that the motor component of the spinally organized flexor reflex circuitry may also be sensitized by secondary neuroinflammation, the THS-induced bilateral mechanical pain hypersensitivity is likely to involve the ventral horn motor component as well although direct evidence is not available in this study.

## Conclusion

Our present study demonstrated that: (1) THS can induce secondary neuronal death and neuroinflammation in the spinal dorsal horn through axonal retrograde degeneration of the STT projecting system; (2) SDF1-CXCR4 signaling mediates secondary spinal neuroinflammation by glial–glial and glial–neuronal interactions; (3) targeting the secondary spinal neuroinflammation by inhibiting activations of either microglia or astrocytes or by antagonizing CXCR4 can relieve the THS-induced pain hypersensitivity. These results are of particular importance for providing a novel therapeutic route at the spinal level to treat intractable CPSP in clinic.

## Data availability statement

The original contributions presented in the study are included in the article/Supplementary material, further inquiries can be directed to the corresponding author/s.

## Ethics statement

The animal study was reviewed and approved by Institutional Animal Care and Use Committee at the Fouth Military Medical University.

## Author contributions

TL and JC conceived, designed, and contributed to the whole process of the study. X-FC conducted spinal MEA recordings. YYa, FeY, YYu, FaY, and J-LW partially involved in the behavioral assays. X-LW assisted in the preparation of animal model. TL, WS, FeY, and JC wrote the manuscript. All authors contributed to the article and approved the submitted version.

## Footnotes

1
https://github.com/kwikteam/phy

## Supplementary material

Supplementary Figure 1 TdT-mediated dUTP nick end labeling (TUNEL)-labeled cellular profiles and immunofluorescent images of Iba1- and GFAP-labeling in both dorsal and ventral horn of the spinal cord. **(A)** A representative fluorescent image showing coronal view of the whole lumbar spinal cord of TUNEL-labeling (left panel) and quantitative analysis of TUNEL-labeled fluorescent intensity between bilateral dorsal horns 7 days after ITC (*n* = 3 rats) (right panel). **p* < 0.05, contralateral vs. ipsilateral. Scale bar = 200 μm. **(B,C)** Representative immunofluorescent images and intensities of Iba1- and GFAP-labeling in both dorsal and ventral horns of the contralateral spinal cord to ITS or ITC (left panel) and quantitative analysis of immunofluorescent intensities of Iba1- and GFAP-labeling in the ventral horn between ITC and ITS 7 days after treatment (right panel). **p* < 0.05, ***p* < 0.01, ITC vs. ITS. cc, central canal, DC, dorsal column, DH, dorsal horn, LC, lateral column, VC, ventral column, VH, ventral horn.

## Abbreviations

ACC: anterior cingulate cortex
ACF: artificial cerebrospinal fluid
CPSP: central post-stroke pain
CNS: central nervous system
DAPI: 2-(4-amidinophenyl)-6-indolecarbamidine dihydrochloride
EM: electron microscopy
ICD-11: 11th revision of the International Classification of Diseases
FC: fluorocitrate
i.p.: intraperitoneal
i.t.: intrathecal
ITC: intra-thalamic collagenase injection
ITS: intra-thalamic saline injection
LTM: low-threshold mechanoreceptor
MC: minocycline
MEA: multi-electrode array
NS: nociceptive-specific
PBS: phosphate-buffered saline
PBST: PBS with 0.05% Tween 20
Po: posterior nucleus of thalamus
PVDF: polyvinylidene difluoride
PWMT: paw withdrawal mechanical threshold
SDF1: stromal cell-derived factor 1
SDS: sodium dodecyl sulfate
SSRI: selective serotonin reuptake inhibitor
STT: spinothalamic tract
TUNEL: TdT-mediated dUTP nick end labeling
VBC: ventrobasal complex
VPL: ventralis posterolateral nucleus of the thalamus
WDR: wide dynamic range.
