# Supplementary material for: Secondary damage and neuroinflammation in the spinal dorsal horn mediate post-thalamic hemorrhagic stroke pain hypersensitivity: SDF1-CXCR4 signaling mediation
Source: Front Mol Neurosci. 2022 Aug 12;15:911476. doi: 10.3389/fnmol.2022.911476 (PMC9416701; doi:10.3389/fnmol.2022.911476)
Supplement: Supplementary file 1 [file Table_1.DOCX]

Table S1 Key resources of reagents and software used in the current study

| Reagent or resource | Source | Identifier |
| --- | --- | --- |
| Antibody | |  |
| Anti mouse NeuN antibody | Abcam, UK | Cat# 177487 |
| Anti rabbit Bax antibody | Abcam, UK | Cat# sc-7480 |
| Anti rabbit Bcl-2 antibody | Abcam, UK | Cat# sc-492 |
| DAPI | Sigma-Aldrich, USA | Cat# D4952 |
| Anti goat Iba1 antibody | Abcam, UK | Cat# ab5076 |
| Anti mouse GFAP antibody | Millipore, USA | Cat# MAB360 |
| Anti mouse SDF1 | Santa Cruz, USA | Cat# sc-74271 |
| Anti rabbit CXCR4 | Alomone, [Israel](javascript:;) | Cat# ACR-014-AG |
| Anti β-actin antibody | Sigma-Aldrich, USA | Cat# A1978 |
| Anti mouse IgG FITC antibody | Sigma-Aldrich, USA | Cat# F9137 |
| Anti mouse IgG HRP antibody | ZSGB-BIO, China | Cat# ZB2305 |
| Anti rabbit IgG Cy3 antibody | Sigma-Aldrich, USA | Cat# C2306 |
| Drugs |  |  |
| Minocycline | Sigma-Aldrich, USA | Cat# M9511 |
| Fluorocitrate | Sigma-Aldrich, USA | Cat# F9634 |
| AMD3100 | Sigma-Aldrich, USA | Cat# [A5602](https://www.sigmaaldrich.cn/CN/zh/product/sigma/a5602) |
| Critical Commercial Assays | |  |
| [BCA Protein Assay Kit](https://www.thermofisher.com/order/catalog/product/A53225) | Thermo Fisher, USA | Cat# A53225 |
| TUNEL [Kit](https://www.thermofisher.com/order/catalog/product/A53225) | Roche, [Switzerland](javascript:;) | Cat# 684 795 910 |
| Experimental Models: Organisms/Strains | |  |
| Rat: (Sprague Dawley, SD) | Laboratory Animal Center of the Fourth Military Medical University | N/A |
| Software and Algorithms | |  |
| Statistical software | <https://www.ibm.com/nl-en/products/>spss-modeler/ | SPSS 25.0 |
| Image J | National Institutes of Health | 1.47v |
| GraphPad Prism | GraphPad | 8.0 |
| AlphaImager EP | Cell Biosciences. Inc | 3.2.2 |
| FV10-ASW | Olympus Corp.,Ltd | 04.02.02.09 |
| In vivo MEA recording software | PlexControl |  |
| In vivo MEA recording analysis software | https://github.com/kwikteam/phy | Kilosort, [Phy](http://phy.readthedocs.org/en/latest/) |
