# Supplementary material for: Secondary damage and neuroinflammation in the spinal dorsal horn mediate post-thalamic hemorrhagic stroke pain hypersensitivity: SDF1-CXCR4 signaling mediation
Source: Front Mol Neurosci. 2022 Aug 12;15:911476. doi: 10.3389/fnmol.2022.911476 (PMC9416701; doi:10.3389/fnmol.2022.911476)
Supplement: Supplementary file 2 [file Table_2.DOCX]

Table S2 Number of samples, normality and equal variance tests, statistical analytical methods and p values

| **Figure number** | **Number of animals or samples** | **Statistic method** | **Between-sample effects and within-time effects** | **P value with post hoc correction** |
| --- | --- | --- | --- | --- |
| 1B | n =44 rats for ITS  n =59 rats for ITC | Two-way ANOVA RM with Bonferroni post hoc correction | *p* < 0.001  *F_A_* = 54.859  Mauchly's Test of Sphericity  𝜒2 = 171,363  *p* < 0.001 (failed)  then,  Greenhouse-Geisser: *p* < 0.001  *F_B_* = 136.9  Greenhouse-Geisser: *p* = < 0.001  *F_AB_* = 46.773 | ITS-Ipsil *vs* ITC-Ipsil  *p* < 0.001  ITS-Contrl *vs* ITC- Contrl  *p* < 0.001  ITS-Ipsil *vs* ITS-Contrl  *p* =1.000  ITC-Ipsil *vs* ITC-Contrl  *p* =1.000  -1day  ITS-Contrl *vs* ITS-Ipsil *p* =1.000  ITC-Contrl *vs* ITC-Ipsil  *p* =1.000  ITS-Contrl *vs* ITC- Contrl *p* =0.667  ITS-Ipsil *vs* ITC- Ipsil  *p* =0.187  3day  ITS-Contrl *vs* ITS-Ipsil *p* =1.000  ITC-Contrl *vs* ITC-Ipsil  *p* =1.000  ITS-Contrl *vs* ITC- Contrl *p*< 0.001  ITS-Ipsil *vs* ITC- Ipsil  *p* < 0.001  7day  ITS-Contrl *vs* ITS-Ipsil *p* =1.000  ITC-Contrl *vs* ITC-Ipsil  *p* =1.000  ITS-Contrl *vs* ITC- Contrl *p*< 0.001  ITS-Ipsil *vs* ITC- Ipsil  *p* < 0.001  7day  ITS-Contrl *vs* ITS-Ipsil *p* =1.000  ITC-Contrl *vs* ITC-Ipsil  *p* =1.000  ITS-Contrl *vs* ITC- Contrl *p*< 0.001  ITS-Ipsil *vs* ITC- Ipsil  *p* < 0.001  ITC -1day *vs 3 day p* < 0.001  ITC -1day *vs 7 day p* < 0.001  ITC -1day *vs 10 day p* < 0.001  ITC 3day *vs 7 day p* < 0.001  ITC 3day *vs 10 day p* < 0.001  ITC 7day *vs 10 day p =* 0.097 |

| **Figure number** | **Number of animals or samples** | **Normality test or equal variance test** | **Statistic method** | ***p* value** | ***t* value** |
| --- | --- | --- | --- | --- | --- |
| 2B | n =3 rats for ITS  n =3 rats for ITC |  | *U* -test | *p* <0.001 | *U* =107.000 |
| 2D | n =3 rats for ITS  n =3 rats for ITC |  | *U* -test | *p* <0.001 | *U* =436.000 |
| 2F | n =3 rats for ITS  n =3 rats for ITC |  | *U*- test | *p* <0.001 | *U* = 2.000 |
| 2G  Contrl Bcl-2 | n =5 rats for ITS  n =5 rats for ITC | passed/passed | *t-* test | *p* =0.007 | *t* =3.572 |
| 2G  Ipsil Bcl-2 | n =5 rats for ITS  n =5 rats for ITC | passed/passed | *t-* test | *p* =0.047 | *t* =2.345 |
| 2H  Contrl Bax | n =5 rats for ITS  n =5 rats for ITC | passed/passed | *t-* test | *p* =0.004 | *t* =-3.942 |
| 2H  Ipsil Bax | n =5 rats for ITS  n =5 rats for ITC | passed/passed | *t-* test | *p* =0.016 | *t* =-3.026 |
|  | | | | | |
| 3C  NC | n =3 rats for ITS  n =3 rats for ITC | passed/passed | *t-* test | *p* =0.004 | *t* =3.374 |
| 3C  DC | n =3 rats for ITS  n =3 rats for ITC | passed/failed | *t-* test | *p* =0.003 | *t* =-4.177 |
|  | | | | | |
| 5B Iba-1 | n =3 rats for ITS  n =3 rats for ITC |  | *U-* test | *p* <0.001 | *U* =234.000 |
| 5B GFAP | n =3 rats for ITS  n =3 rats for ITC |  | *U-* test | *p* <0.001 | *U* =92.000 |
| 5B SDF1 | n =3 rats for ITS  n =3 rats for ITC |  | *U-* test | *p* <0.001 | *U* =149.000 |
| 5B CXR4 | n =3 rats for ITS  n =3 rats for ITC |  | *U-* test | *p* <0.001 | *U* =198.500 |
| 5D | n =3 rats |  | *Kruskal-Wallis* test | *p* =0.061 | *H* =5.600 |
| 5F  Ipsil Iba-1 | n =5 rats for ITS  n =5 rats for ITC | passed/passed | *t-* test | *p* =0.021 | *t* =-2.856 |
| 5F  Ipsil GFAP | n =5 rats for ITS  n =5 rats for ITC | passed/passed | *t-* test | *p* =0.002 | *t* =-4.596 |
| 5F  Ipsil SDF1 | n =5 rats for ITS  n =5 rats for ITC | failed | *U-* test | *p* =0.009 | *U <0.001* |
| 5F  Ipsil CXCR4 | n =5 rats for ITS  n =5 rats for ITC | passed/passed | *t-* test | *p* =0.010 | *t* =-3.352 |
| 5F  Contrl Iba-1 | n =5 rats for ITS  n =5 rats for ITC | passed/passed | *t-* test | *p* =0.011 | *t =-3.284* |
| 5F  Contrl GFAP | n =5 rats for ITS  n =5 rats for ITC | passed/passed | *t-* test | *p* =0.035 | *t* =-2.534 |
| 5F  Contrl SDF1 | n =5 rats for ITS  n =5 rats for ITC | passed/passed | *t-* test | *p* =0.032 | *t* =-2.594 |
| 5F  Contrl CXCR4 | n =5 rats for ITS  n =5 rats for ITC | passed/passed | *t-* test | *p* =0.025 | *t* =-2.746 |

| **Figure number** | **Number of animals or samples** | **Normality test or equal variance test** | **Statistic method 1** | **post hoc multiple comparisons test** |
| --- | --- | --- | --- | --- |
| 7B Iba-1 | n =5 rats for ITS  n =5 rats for ITC | passed/passed | One-way ANOVA test  F=3.817  *p* =0.018 | LSD post hoc test  ITS *vs* ITC-Veh *p* =0.008  *ITC-Veh vs ITC-MC p* =0.040  *ITC-Veh vs ITC-FC p* =0.002  *ITC-Veh vs ITC-AMD p* =0.006 |
| 7B GFAP | n =5 rats for ITS  n =5 rats for ITC | passed/passed | One-way ANOVA test  F=32.343  *p* <0.001 | LSD post hoc test  ITS *vs* ITC-Veh *p* =0.005  *ITC-Veh vs ITC-MC* *p* <0.001  *ITC-Veh vs ITC-FC p* <0.001  *ITC-Veh vs ITC-AMD p* <0.001 |
| 7C SDF1 | n =5 rats for ITS  n =5 rats for ITC | passed/passed | One-way ANOVA test  F=4.962  *p* =0.006 | LSD post hoc test  ITS *vs* ITC-Veh *p* =0.024  *ITC-Veh vs ITC-MC p* =0.020  *ITC-Veh vs ITC-FC p* =0.002  *ITC-Veh vs ITC-AMD p* =0.001 |
| 7C CXCR4 | n =5 rats for ITS  n =5 rats for ITC | passed/passed | One-way ANOVA test  F=13.165  *p* <0.001 | LSD post hoc test  ITS *vs* ITC-Veh *p* =0.001  *ITC-Veh vs ITC-MC p* <0.001  *ITC-Veh vs ITC-FC p* <0.001  *ITC-Veh vs ITC-AMD p* <0.001 |

| **Figure number** | **Number of animals or samples** | **Statistic method 1** | **Between-sample effects and within-time effects** | **Statistic method 2 and *p* values** |
| --- | --- | --- | --- | --- |
| 8A  ITC-Veh *vs* ITC-MC | n= 12 rats for ITC-Veh  n= 11 rats for ITC-MC | Two-way ANOVA RM with Bonferroni post hoc correction | *p* < 0.001  *F_A_* = *F*_0.05, (1,21)_ = 20.869  Mauchly's Test of Sphericity  𝜒^2^ = 69.312  𝜈 = 27  *p* < 0.001 (failed)  then,  Greenhouse-Geisser: *p* < 0.001  *F_B_* = *F*_0.05, (3.633,76.297)_ = 30.853  Greenhouse-Geisser: *p* < 0.001  *F_AB_* = *F*_0.05, (3.633,76.297)_ = 8.096 | Friedman′s *M* test  ITC-Veh: *p* < 0.001  ITC-MC: *p* < 0.001  *U* test  ITC-Veh *vs* ITC-MC (pre): *p* = 0.379  ITC-Veh *vs* ITC-MC (post): *p* = 0.079  ITC-Veh *vs* ITC-MC (2 h): *p* < 0.001  ITC-Veh *vs* ITC-MC (4 h): *p* < 0.001  ITC-Veh *vs* ITC-MC (6 h): *p* = 0.013  ITC-Veh *vs* ITC-MC (8 h): *p* = 0.009  ITC-Veh *vs* ITC-MC (24 h): *p* = 0.211  ITC-Veh *vs* ITC-MC (48 h): *p* = 0.449 |
| 8A  ITS-Veh *vs* ITS-MC | n= 12 rats for ITS-Veh  n= 5 rats for ITS-MC | Two-way ANOVA RM with Bonferroni post hoc correction | *p =0.516*  *F_A_ =0.444*  Mauchly's Test of Sphericity  *p < 0.001(failed)*  *then,* Greenhouse-Geisser:  *p =0.881*  *F_B_ = 0.242*  *P= 0.797*  *F_AB_ = 0.462* |  |
| 8B  ITC-Veh *vs* ITC-MC | n= 12 rats for ITC-Veh  n= 11 rats for ITC-MC | Two-way ANOVA RM with Bonferroni post hoc correction | *p* = 0.025  *F_A_* = *F*_0.05, (1,21)_ = 5.842  Mauchly's Test of Sphericity  𝜒^2^ = 126.718  𝜈 = 27  *p* < 0.001 (failed)  then,  Greenhouse-Geisser: *p* < 0.001  *F_B_* = *F*_0.05, (2.478,52.046)_ = 19.604  Greenhouse-Geisser: *p* = 0.008  *F_AB_* = *F*_0.05, (2.478,52.046)_ = 4.781 | Friedman′s *M* test  ITC-Veh: *p* < 0.001  ITC-MC: *p* < 0.001  *U* test  ITC-Veh *vs* ITC-MC (pre): *p* = 0.695  ITC-Veh *vs* ITC-MC (post): *p* = 0.976  ITC-Veh *vs* ITC-MC (2 h): *p* = 0.009  ITC-Veh *vs* ITC-MC (4 h): *p* = 0.032  ITC-Veh *vs* ITC-MC (6 h): *p* = 0.069  ITC-Veh *vs* ITC-MC (8 h): *p* = 0.118  ITC-Veh *vs* ITC-MC (24 h): *p* = 0.566  ITC-Veh *vs* ITC-MC (48 h): *p* = 1.000 |
| 8B  ITS-Veh *vs* ITS-MC | n= 12 rats for ITS-Veh  n= 5 rats for ITS-MC | Two-way ANOVA RM with Bonferroni post hoc correction | *p =0.589*  *F_A_ =0.305*  Mauchly's Test of Sphericity  *p < 0.001(failed)*  *then,* Greenhouse-Geisser:  *p =0.744*  *F_B_ = 0.421*  *P= 0.322*  *F_AB_ = 1.197* |  |
| 8C  ITC-Veh *vs* ITC-FC | n= 12 rats for ITC-Veh  n= 10 rats for ITC-FC | Two-way ANOVA RM with Bonferroni post hoc correction | *p* = 0.011  *F_A_* = *F*_0.05, (1,20)_ = 7.825  Mauchly's Test of Sphericity  𝜒^2^ = 89.204  𝜈 = 27  *p* < 0.001 (failed)  then,  Greenhouse-Geisser: *p* < 0.001  *F_B_* = *F*_0.05, (3.141,62.817)_ = 22.786  Greenhouse-Geisser: *p* = 0.029  *F_AB_* = *F*_0.05, (3.141,62.817)_ = 3.167 | Friedman′s M test  ITC-Veh: *p* < 0.001  ITC-FC: *p* < 0.001  U test  ITC-Veh *vs* ITC-FC (pre): *p* = 0.674  ITC-Veh *vs* ITC-FC (post): *p* = 0.497  ITC-Veh *vs* ITC-FC (2 h): *p* = 0.007  ITC-Veh *vs* ITC-FC (4 h): *p* = 0.002  ITC-Veh *vs* ITC-FC (6 h): *p* = 0.004  ITC-Veh *vs* ITC-FC (8 h): *p* = 0.159  ITC-Veh *vs* ITC-FC (24 h): *p* = 0.418  ITC-Veh *vs* ITC-FC (48 h): *p* = 0.771 |
| 8C  ITS-Veh *vs* ITS-FC | n= 12 rats for ITS-Veh  n= 6 rats for ITS-FC | Two-way ANOVA RM with Bonferroni post hoc correction | *P=0.551*  *F_A_ = 0.331*  Mauchly's Test of Sphericity  *p < 0.001(failed)*  *then,* Greenhouse-Geisser  *p =0.814*  *F_B_ = 0.3549*  *p =0.826*  *F_AB_ = 0.280* |  |
| 8D  ITC-Veh *vs* ITC-FC | n= 12 rats for ITC-Veh  n= 10 rats for ITC-FC | Two-way ANOVA RM with Bonferroni post hoc correction | *p* = 0.043  *F_A_* = *F*_0.05, (1,20)_ =4.71  Mauchly's Test of Sphericity  𝜒^2^ = 122.969  𝜈 = 27  *p* < 0.001 (failed)  then,  Greenhouse-Geisser: *p* < 0.001  *F_B_* = *F*_0.05, (2.696,53.922)_ = 34.797  Greenhouse-Geisser: *p* = 0.008  *F_AB_* = *F*_0.05, (2.696,53.922)_ = 6.061 | Friedman′s *M* test  ITC-Veh: *p* < 0.001  ITC-FC: *p* < 0.001  *U* test  ITC-Veh *vs* ITC-FC (pre): *p* = 0.974  ITC-Veh *vs* ITC-FC (post): *p* = 0.722  ITC-Veh *vs* ITC-FC (2 h): *p* = 0.050  ITC-Veh *vs* ITC-FC (4 h): *p* = 0.014  ITC-Veh *vs* ITC-FC (6 h): *p* = 0.009  ITC-Veh *vs* ITC-FC (8 h): *p* = 0.497  ITC-Veh *vs* ITC-FC (24 h): *p* = 0.722  ITC-Veh *vs* ITC-FC (48 h): *p* = 0.771 |
| 8D  ITS-Veh *vs* ITS-FC | n= 12 rats for ITS-Veh  n= 6 rats for ITS-FC | Two-way ANOVA RM with Bonferroni post hoc correction | *P=0.521*  *F_A_ = 0.432*  Mauchly's Test of Sphericity  *p < 0.001(failed)*  *then,* Greenhouse-Geisser  *p =0.702*  *F_B_ = 0.477*  *p =0.736*  *F_AB_ = 0.425* |  |
| 8E  ITC-Veh *vs* ITC-AMD | n= 12 rats for ITC-Veh  n= 10 rats for ITC-AMD | Two-way ANOVA RM with Bonferroni post hoc correction | *p* = 0.001  *F_A_* = *F*_0.05, (1,20)_ = 15.795  Mauchly's Test of Sphericity  𝜒^2^ = 118.724  𝜈 = 27  *p* < 0.001 (failed)  then,  Greenhouse-Geisser: *p* < 0.001  *F_B_* = *F*_0.05, (2.347,46.936)_ = 64.337  Greenhouse-Geisser: *p* < 0.001  *F_AB_* = *F*_0.05, (2.347,46.936)_ = 11.394 | Friedman′s *M* test  ITC-Veh: *p* < 0.001  ITC-AMD: *p* < 0.001  *U* test  ITC-Veh vs ITC-AMD (pre): *p* = 0.228  ITC-Veh vs ITC-AMD (post): *p* = 0.582  ITC-Veh vs ITC-AMD (2 h): *p* = 0.021  ITC-Veh vs ITC-AMD (4 h): *p* < 0.001  ITC-Veh vs ITC-AMD (6 h): *p* < 0.001  ITC-Veh vs ITC-AMD (8 h): *p* < 0.001  ITC-Veh vs ITC-AMD (24 h): *p* = 0.923  ITC-Veh vs ITC-AMD (48 h): *p* = 0.821 |
| 8E  ITS-Veh *vs* ITS-AMD | n= 12 rats for ITS-Veh  n= 5 rats for ITS-AMD | Two-way ANOVA RM with Bonferroni post hoc correction | *P=0.909*  *F_A_ = 0.013*  Mauchly's Test of Sphericity  *p < 0.001(failed)*  *then,* Greenhouse-Geisser  *p =0.458*  *F_B_ = 0.915*  *p =0.475*  *F_AB_ = 0.885* |  |
| 8F  ITC-Veh *vs* ITC-AMD | n= 12 rats for ITC-Veh  n= 10 rats for ITC-AMD | Two-way ANOVA RM with Bonferroni post hoc correction | *p* = 0.039  *F_A_* = *F*_0.05, (1,20)_ = 4.873  Mauchly's Test of Sphericity  𝜒^2^ = 134.690  𝜈 = 27  *p* < 0.001 (failed)  then,  Greenhouse-Geisser: *p* < 0.001  *F_B_* = *F*_0.05, (2.291,45.824)_ = 65.620  Greenhouse-Geisser: *p* < 0.001  *F_AB_* = *F*_0.05, (2.291,45.824)_ = 13.303 | Friedman′s *M* test  IV: *p* < 0.001  IA: *p* < 0.001  *U* test  ITC-Veh vs ITC-AMD (pre): *p* = 0.093  ITC-Veh vs ITC-AMD (post): *p* = 0.582  ITC-Veh vs ITC-AMD (2 h): *p* = 0.418  ITC-Veh vs ITC-AMD (4 h): *p* = 0.001  ITC-Veh vs ITC-AMD (6 h): *p* < 0.001  ITC-Veh vs ITC-AMD (8 h): *p* = 0.003  ITC-Veh vs ITC-AMD (24 h): *p* = 0.722  IV vs IA (48 h): *p* = 0.722 |
| 8F  ITS-Veh *vs* ITS-AMD | n= 12 rats for ITS-Veh  n= 5 rats for ITS-AMD | Two-way ANOVA RM with Bonferroni post hoc correction | *P=0.945*  *F_A_ = 0.003*  Mauchly's Test of Sphericity  *p < 0.001(failed)*  *then,* Greenhouse-Geisser  *p =0.497*  *F_B_ = 0.845*  *p =0.595*  *F_AB_ = 0.688* |  |
