# Supplementary material for: Secondary damage and neuroinflammation in the spinal dorsal horn mediate post-thalamic hemorrhagic stroke pain hypersensitivity: SDF1-CXCR4 signaling mediation
Source: Front Mol Neurosci. 2022 Aug 12;15:911476. doi: 10.3389/fnmol.2022.911476 (PMC9416701; doi:10.3389/fnmol.2022.911476)

Table S3 Comparisons of non-normalized (raw) and normalized quantitative data of Western blot results from each animal or sample, and graphs plotted with raw data of paw withdrawal mechanical threshold (PWMT, g) in response to von Frey filament stimuli

Fig. 2G

| Non-normalized |  | Rat1 | Rat2 | Rat3 | Rat4 | Rat5 |
| --- | --- | --- | --- | --- | --- | --- |
| Contral-Bcl-2/Actin | ITS | 1.76678 | 1.193597 | 1.630426 | 1.205662 | 1.627955 |
|  | ITC | 1.035372 | 1.085862 | 0.808867 | 1.175841 | 0.818722 |
| Ipsil- Bcl-2/Actin | ITS | 1.14431 | 1.109871 | 0.697587 | 1.049942 | 1.382633 |
|  | ITC | 0.832595 | 0.710821 | 0.584804 | 0.873081 | 0.911284 |
|  |  |  |  |  |  |  |
| Normalized |  |  |  |  |  |  |
| Contral- Bcl-2/Actin | ITS | 1.189844 | 0.803832 | 1.098016 | 0.811957 | 1.096351 |
|  | ITC | 0.697274 | 0.731277 | 0.544734 | 0.791874 | 0.551371 |
| Ipsil- Bcl-2/Actin | ITS | 1.062628 | 1.030647 | 0.647792 | 0.974995 | 1.283939 |
|  | ITC | 0.773163 | 0.660081 | 0.54306 | 0.810759 | 0.846235 |

Fig. 2H

| Non-normalized |  | Rat1 | Rat2 | Rat3 | Rat4 | Rat5 |
| --- | --- | --- | --- | --- | --- | --- |
| Contral-Bax/Actin | ITS | 0.725217 | 0.532196 | 0.61552 | 0.573575 | 0.685216 |
|  | ITC | 1.203707 | 1.03048 | 0.947798 | 0.722837 | 0.918786 |
| Ipsil- Bax /Actin | ITS | 0.756231 | 0.835666 | 0.591081 | 0.790158 | 0.532372 |
|  | ITC | 0.864221 | 1.103497 | 0.961472 | 1.249411 | 0.820145 |
|  |  |  |  |  |  |  |
| Normalized |  |  |  |  |  |  |
| Contral- Bax /Actin | ITS | 1.157856 | 0.849686 | 0.982718 | 0.915749 | 1.093991 |
|  | ITC | 1.921796 | 1.645228 | 1.513221 | 1.154056 | 1.466901 |
| Ipsil- Bax /Actin | ITS | 1.078633 | 1.191933 | 0.843075 | 1.127023 | 0.759336 |
|  | ITC | 1.232661 | 1.573947 | 1.371373 | 1.782068 | 1.169794 |

Bax expression

✱✱

✱

✱✱

✱

Bcl-2 expression

Graphs showing averaged protein expressions from non-normalized raw western blot bands with particular reference to Fig.2G-H (n=5 rats).

Fig. 5F

| Non-normalized |  | Rat1 | Rat2 | Rat3 | Rat4 | Rat5 |
| --- | --- | --- | --- | --- | --- | --- |
| Contral-SDF1/Actin | ITS | 0.264810072 | 0.897167485 | 0.385949879 | 0.609914853 | 1.00345944 |
|  | ITC | 0.856167277 | 1.53181187 | 0.875240634 | 1.280309592 | 1.094835592 |
| Ipsil-SDF1/Actin | ITS | 0.270991368 | 0.950857743 | 0.758202204 | 0.886226217 | 0.866901033 |
|  | ITC | 1.004571682 | 1.022174806 | 1.058098201 | 1.568769983 | 0.986842157 |
|  |  |  |  |  |  |  |
| Normalized |  |  |  |  |  |  |
| Contral-SDF1/Actin | ITS | 0.418830746 | 1.41898427 | 0.610428729 | 0.964657766 | 1.58709849 |
|  | ITC | 1.354137235 | 2.42275493 | 1.384304172 | 2.02497215 | 1.731621474 |
| Ipsil-SDF1/Actin | ITS | 0.362949915 | 1.273522986 | 1.015491479 | 1.186959318 | 1.161076303 |
|  | ITC | 1.34546428 | 1.36904087 | 1.417154554 | 2.101118331 | 1.321718396 |

| Non-normalized |  | Rat1 | Rat2 | Rat3 | Rat4 | Rat5 |
| --- | --- | --- | --- | --- | --- | --- |
| Contra-Iba-1/Actin | ITS | 0.649224525 | 0.619866177 | 0.291644749 | 0.818201303 | 0.356074483 |
|  | ITC | 1.020950512 | 0.95144082 | 0.8170089 | 1.004691896 | 0.759451549 |
| Ipsil-Iba-1/Actin | ITS | 0.716318053 | 0.775466939 | 0.524777687 | 0.798032855 | 0.500647196 |
|  | ITC | 1.04121403 | 0.901155583 | 1.508948884 | 1.035887025 | 0.792347236 |
|  |  |  |  |  |  |  |
| Normalized |  |  |  |  |  |  |
| Contral-Iba-1/Actin | ITS | 1.186877253 | 1.133205906 | 0.533169197 | 1.495791483 | 0.650956161 |
|  | ITC | 1.86644665 | 1.739372781 | 1.493611596 | 1.836723524 | 1.388388352 |
| Ipsil-Iba-1/Actin | ITS | 1.080340282 | 1.169547755 | 0.791461938 | 1.203581337 | 0.755068688 |
|  | ITC | 1.570343584 | 1.359109508 | 2.275774366 | 1.562309473 | 1.195006369 |

| Non-normalized |  | Rat1 | Rat2 | Rat3 | Rat4 | Rat5 |
| --- | --- | --- | --- | --- | --- | --- |
| Contral-CXCR4/Actin | ITS | 0.650330973 | 0.910754957 | 0.913500403 | 0.829618015 | 0.232840154 |
|  | ITC | 1.160981568 | 1.096890371 | 1.126419564 | 1.479929541 | 0.875596449 |
| Ipsil-CXCR4/Actin | ITS | 0.937786942 | 0.908361008 | 0.854431347 | 0.734629097 | 0.374006975 |
|  | ITC | 1.072846365 | 1.254518862 | 0.997274577 | 1.500844333 | 1.244444759 |
|  |  |  |  |  |  |  |
| Normalized |  |  |  |  |  |  |
| Contral-CXCR4/Actin | ITS | 0.919314095 | 1.287451934 | 1.291332923 | 1.172755976 | 0.329145072 |
|  | ITC | 1.641174669 | 1.550574739 | 1.592317489 | 2.092042579 | 1.237751531 |
| Ipsil-CXCR4/Actin | ITS | 1.230945026 | 1.19232036 | 1.121531949 | 0.964278763 | 0.490923903 |
|  | ITC | 1.408224872 | 1.646689331 | 1.309028869 | 1.97001769 | 1.633466 |

| Non-normalized |  | Rat1 | Rat2 | Rat3 | Rat4 | Rat5 |
| --- | --- | --- | --- | --- | --- | --- |
| Contral-GFAP/Actin | ITS | 0.746017329 | 1.037026193 | 0.173411935 | 0.527080408 | 0.379643545 |
|  | ITC | 1.073975065 | 1.269294987 | 1.229954022 | 1.018450013 | 0.636824991 |
| Ipsil-GFAP/Actin | ITS | 0.870700616 | 0.390364265 | 0.319706598 | 0.611467919 | 0.547406414 |
|  | ITC | 1.085148798 | 1.381823156 | 1.163691199 | 0.932376526 | 1.001335384 |
|  |  |  |  |  |  |  |
| Normalized |  |  |  |  |  |  |
| Contral-GFAP/Actin | ITS | 1.302777825 | 1.810969633 | 0.302831067 | 0.920446002 | 0.662975473 |
|  | ITC | 1.875493833 | 2.216583045 | 2.147881509 | 1.778529857 | 1.112094109 |
| Ipsil-GFAP/Actin | ITS | 1.589075151 | 0.712435642 | 0.583481625 | 1.115961627 | 0.999045955 |
|  | ITC | 1.980454541 | 2.521901097 | 2.123798621 | 1.701636982 | 1.827490583 |


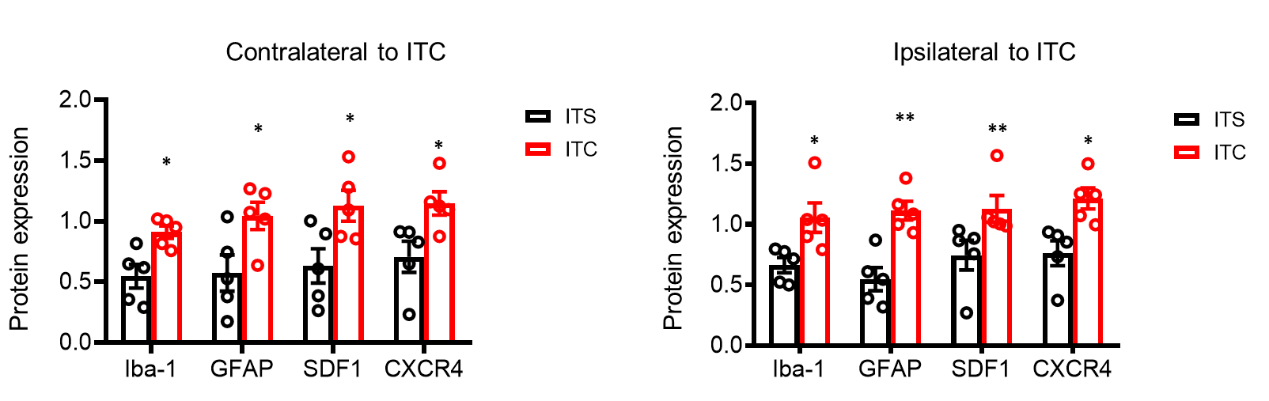


Graphs showing averaged protein expressions from non-normalized raw western blot bands with particular reference to Fig.5F (n=5 rats).

Fig. 7B and C

| Non-normalized |  | sample 1 | sample 2 | sample 3 | sample 4 | sample 5 |
| --- | --- | --- | --- | --- | --- | --- |
| SDF1/Actin | ITS | 1.143708503 | 0.943950025 | 1.068221043 | 0.775319295 | 0.37146131 |
|  | ITC | 1.333290221 | 1.515020859 | 1.34159645 | 1.056672608 | 1.171060598 |
|  | ITC-MC | 1.168740816 | 0.982864292 | 0.37771506 | 0.93620087 | 0.77884379 |
|  | ITC-FC | 0.941467067 | 0.915674005 | 0.400028016 | 0.794807046 | 0.258578337 |
|  | ITC-AMD | 0.788519132 | 0.582857735 | 0.185231525 | 0.791388872 | 0.554168861 |
|  |  |  |  |  |  |  |
| Normalized |  |  |  |  |  |  |
| SDF1/Actin | ITS | 1.329071384 | 1.096937692 | 1.241349536 | 0.900976679 | 0.431664709 |
|  | ITC | 1.549378949 | 1.760562996 | 1.559031383 | 1.227929425 | 1.360856482 |
|  | ITC-MC | 1.358160729 | 1.142158864 | 0.438932014 | 1.087932618 | 0.905072395 |
|  | ITC-FC | 1.094052317 | 1.064078927 | 0.464861272 | 0.923622844 | 0.30048659 |
|  | ITC-AMD | 0.916315836 | 0.677322529 | 0.215252329 | 0.919650681 | 0.643983999 |

| Non-normalized |  | sample 1 | sample 2 | sample 3 | sample 4 | sample 5 |
| --- | --- | --- | --- | --- | --- | --- |
| Iba-1/Actin | ITS | 0.934575343 | 0.783982374 | 0.705892822 | 0.817616632 | 0.330305404 |
|  | ITC | 1.491564146 | 1.357963597 | 1.18122666 | 0.982051342 | 1.114111881 |
|  | ITC-MC | 1.191320513 | 1.064939738 | 0.431973952 | 0.737360229 | 0.790558039 |
|  | ITC-FC | 0.944903404 | 0.924398651 | 0.514526658 | 0.399016838 | 0.331707832 |
|  | ITC-AMD | 0.986522635 | 0.642956969 | 0.177783761 | 0.69486958 | 0.982789495 |
|  |  |  |  |  |  |  |
| Normalized |  |  |  |  |  |  |
| Iba-1/Actin | ITS | 1.308059732 | 1.097285289 | 0.987988804 | 1.144360807 | 0.462305368 |
|  | ITC | 2.087637998 | 1.900646655 | 1.653280327 | 1.374508568 | 1.559344466 |
|  | ITC-MC | 1.667407987 | 1.490521657 | 0.604603723 | 1.032031533 | 1.106488787 |
|  | ITC-FC | 1.322515197 | 1.293816129 | 0.720146971 | 0.558475956 | 0.464268249 |
|  | ITC-AMD | 1.380766723 | 0.899901893 | 0.248831495 | 0.972560344 | 1.375541709 |

| Non-normalized |  | sample 1 | sample 2 | sample 3 | sample 4 | sample 5 |
| --- | --- | --- | --- | --- | --- | --- |
| CXCR4/Actin | ITS | 0.97347672 | 0.657510079 | 0.318278351 | 0.965791667 | 0.544559007 |
|  | ITC | 1.046664914 | 1.185394627 | 1.193654548 | 1.048347435 | 1.083976363 |
|  | ITC-MC | 0.544181137 | 0.696798226 | 0.42666814 | 0.632305664 | 0.704754763 |
|  | ITC-FC | 0.470151704 | 0.458043996 | 0.2537279 | 0.299535089 | 0.59691077 |
|  | ITC-AMD | 0.46470343 | 0.446718529 | 0.635722892 | 0.0669624 | 0.309446421 |
|  |  |  |  |  |  |  |
| Normalized |  |  |  |  |  |  |
| CXCR4/Actin | ITS | 1.406914481 | 0.950264584 | 0.459990888 | 1.395807679 | 0.787022367 |
|  | ITC | 1.512689511 | 1.713188237 | 1.725125864 | 1.51512117 | 1.566613777 |
|  | ITC-MC | 0.786476251 | 1.007045668 | 0.616640924 | 0.913837976 | 1.01854483 |
|  | ITC-FC | 0.679485422 | 0.661986791 | 0.366699531 | 0.432902242 | 0.862683604 |
|  | ITC-AMD | 0.67161132 | 0.645618693 | 0.918776714 | 0.096777219 | 0.447226567 |

| Non-normalized |  | sample 1 | sample 2 | sample 3 | sample 4 | sample 5 |
| --- | --- | --- | --- | --- | --- | --- |
| GFAP/Actin | ITS | 0.766185911 | 0.910524697 | 0.509157162 | 1.05895994 | 1.067961682 |
|  | ITC | 1.188561071 | 1.137339498 | 1.183902612 | 1.069508042 | 1.189800976 |
|  | ITC-MC | 0.284518539 | 0.507947937 | 0.390725224 | 0.376977064 | 0.50405413 |
|  | ITC-FC | 0.368239642 | 0.536672328 | 0.191747803 | 0.370318113 | 0.401118106 |
|  | ITC-AMD | 0.247079201 | 0.339094722 | 0.336393994 | 0.335459883 | 0.495193701 |
|  |  |  |  |  |  |  |
| Normalized |  |  |  |  |  |  |
| GFAP/Actin | ITS | 0.888271883 | 1.055609971 | 0.590287533 | 1.227697256 | 1.238133358 |
|  | ITC | 1.377949353 | 1.318566007 | 1.372548604 | 1.239926119 | 1.379386828 |
|  | ITC-MC | 0.329854432 | 0.588885627 | 0.452984354 | 0.437045529 | 0.584371371 |
|  | ITC-FC | 0.426915865 | 0.622187034 | 0.222301376 | 0.429325524 | 0.465033265 |
|  | ITC-AMD | 0.286449417 | 0.39312692 | 0.389995851 | 0.388912897 | 0.5740991 |


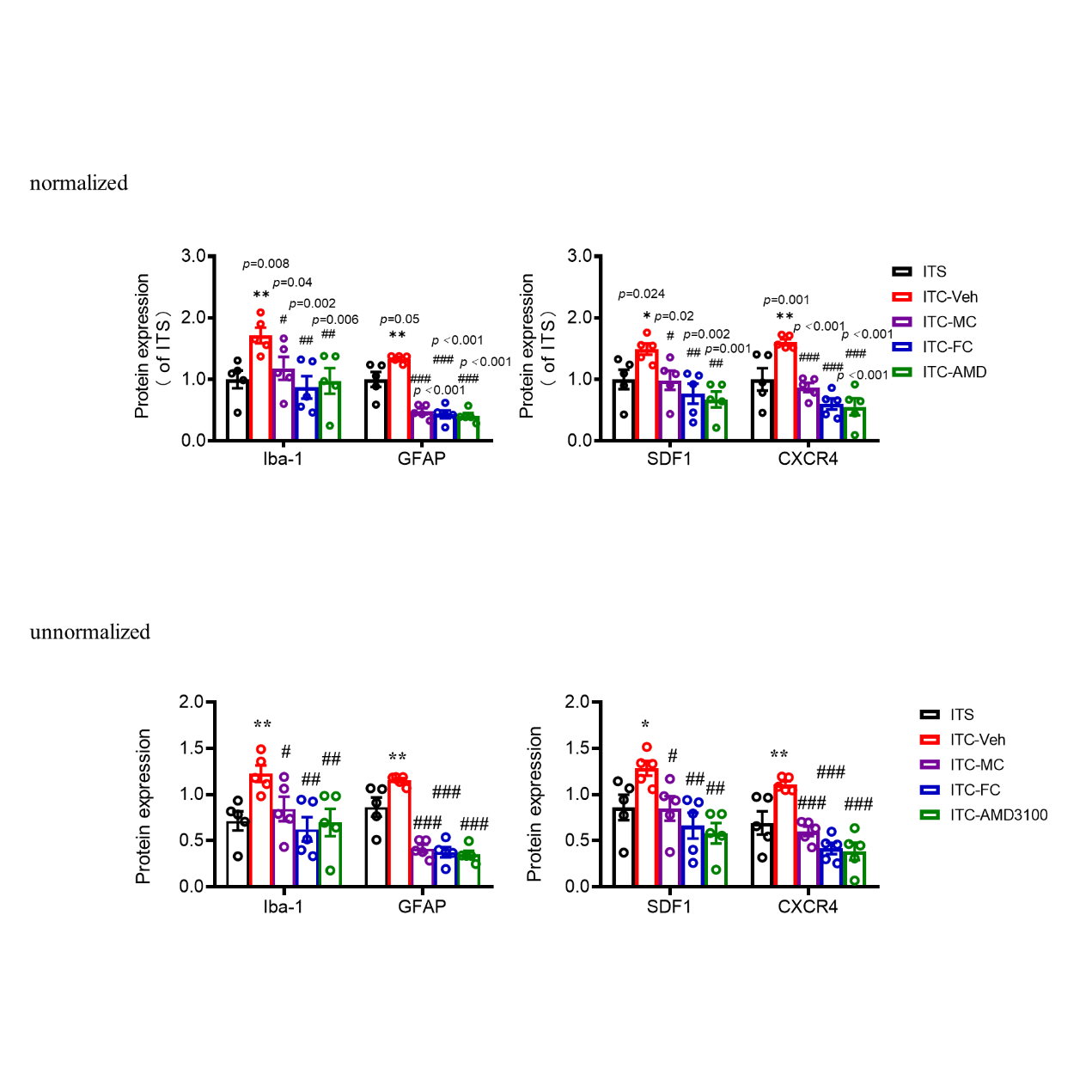


Graphs showing averaged protein expressions from non-normalized raw western blot bands with particular reference to Fig.7B-C (n=5 rats).

Fig.8

Graphs plotted with raw data of paw withdrawal mechanical threshold (PWMT, g) in response to von Frey filament stimuli, with particular reference to Fig.8


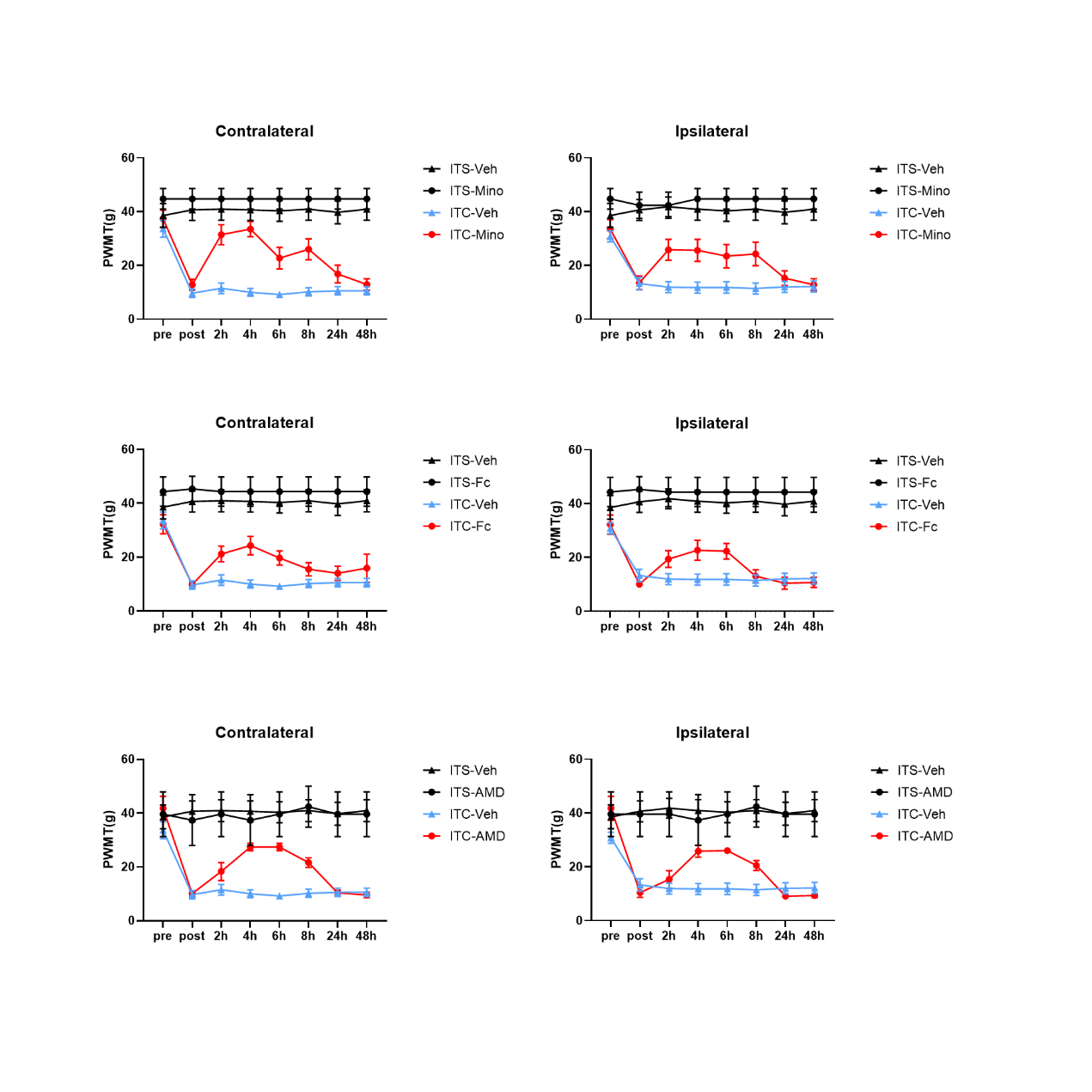

Supplement: Supplementary file 3 [file Table_3.DOCX]
