# Supplementary figures and images for: Secondary damage and neuroinflammation in the spinal dorsal horn mediate post-thalamic hemorrhagic stroke pain hypersensitivity: SDF1-CXCR4 signaling mediation
Source: Front Mol Neurosci. 2022 Aug 12;15:911476. doi: 10.3389/fnmol.2022.911476 (PMC9416701; doi:10.3389/fnmol.2022.911476)

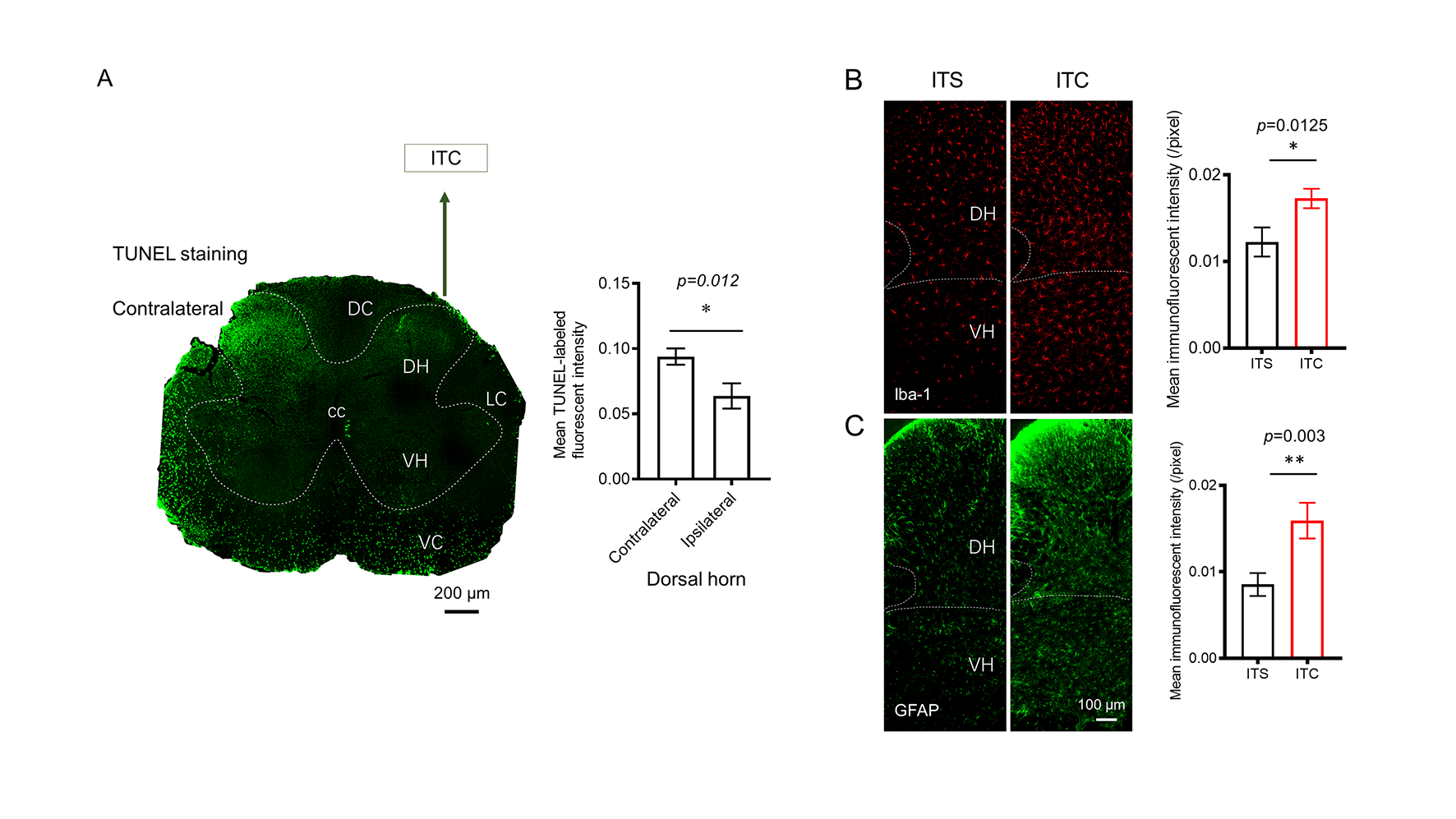

Supplement: Supplementary Figure 1 — TdT-mediated dUTP nick end labeling (TUNEL)-labeled cellular profiles and immunofluorescent images of Iba1- and GFAP-labeling in both dorsal and ventral horn of the spinal cord. (A) A representative fluorescent image showing coronal view of the whole lumbar spinal cord of TUNEL-labeling (left panel) and quantitative analysis of TUNEL-labeled fluorescent intensity between bilateral dorsal horns 7 days after ITC (n = 3 rats) (right panel). *p < 0.05, contralateral vs. ipsilateral. Scale bar = 200 μm. (B,C) Representative immunofluorescent images and intensities of Iba1- and GFAP-labeling in both dorsal and ventral horns of the contralateral spinal cord to ITS or ITC (left panel) and quantitative analysis of immunofluorescent intensities of Iba1- and GFAP-labeling in the ventral horn between ITC and ITS 7 days after treatment (right panel). *p < 0.05, **p < 0.01, ITC vs. ITS. cc, central canal, DC, dorsal column, DH, dorsal horn, LC, lateral column, VC, ventral column, VH, ventral horn. [file Image_1.TIF]
